# Genetic manipulation of putrescine biosynthesis reprograms the cellular transcriptome and the metabolome

**DOI:** 10.1186/s12870-016-0796-2

**Published:** 2016-05-18

**Authors:** Andrew F. Page, Leland J. Cseke, Rakesh Minocha, Swathi A. Turlapati, Gopi K. Podila, Alexander Ulanov, Zhong Li, Subhash C. Minocha

**Affiliations:** Department of Biological Sciences, University of New Hampshire, Durham, NH 03824 USA; Department of Biological Sciences, University of Alabama in Huntsville, Huntsville, AL 35899 USA; USDA Forest Service, Northern Research Station, Durham, NH 03824 USA; Metabolomics Center, Roy J. Carver Biotechnology Center, University of Illinois at Urbana-Champaign, Champaign, IL 61801 USA

**Keywords:** Genetic manipulation, Metabolome, Microarrays, Ornithine decarboxylase, Polyamines, Populus, Transcriptome

## Abstract

**Background:**

With the increasing interest in metabolic engineering of plants using genetic manipulation and gene editing technologies to enhance growth, nutritional value and environmental adaptation, a major concern is the potential of undesirable broad and distant effects of manipulating the target gene or metabolic step in the resulting plant. A comprehensive transcriptomic and metabolomic analysis of the product may shed some useful light in this regard. The present study used these two techniques with plant cell cultures to analyze the effects of genetic manipulation of a single step in the biosynthesis of polyamines because of their well-known roles in plant growth, development and stress responses.

**Results:**

The transcriptomes and metabolomes of a control and a high putrescine (HP) producing cell line of poplar (*Populus nigra* x *maximowiczii*) were compared using microarrays and GC/MS. The HP cells expressed an *ornithine decarboxylase* transgene and accumulated several-fold higher concentrations of putrescine, with only small changes in spermidine and spermine. The results show that up-regulation of a single step in the polyamine biosynthetic pathway (i.e. ornithine → putrescine) altered the expression of a broad spectrum of genes; many of which were involved in transcription, translation, membrane transport, osmoregulation, shock/stress/wounding, and cell wall metabolism. More than half of the 200 detected metabolites were significantly altered (p ≤ 0.05) in the HP cells irrespective of sampling date. The most noteworthy differences were in organic acids, carbohydrates and nitrogen-containing metabolites.

**Conclusions:**

The results provide valuable information about the role of polyamines in regulating nitrogen and carbon use pathways in cell cultures of high putrescine producing transgenic cells of poplar *vs.* their low putrescine counterparts. The results underscore the complexity of cellular responses to genetic perturbation of a single metabolic step related to nitrogen metabolism in plants. Combined with recent studies from our lab, where we showed that higher putrescine production caused an increased flux of glutamate into ornithine concurrent with enhancement in glutamate production via additional nitrogen and carbon assimilation, the results from this study provide guidance in designing transgenic plants with increased nitrogen use efficiency, especially in plants intended for non-food/feed applications (e.g. increased biomass production for biofuels).

**Electronic supplementary material:**

The online version of this article (doi:10.1186/s12870-016-0796-2) contains supplementary material, which is available to authorized users.

## Background

Plant biotechnological endeavors often entail deliberate modification of an organism’s genes and potentially its metabolism for nutritional improvement, better growth, and/or tolerance of abiotic and biotic stresses. In most cases the intent is to target a specific metabolic step with minimal impact on unrelated pathways, thus producing plants, which are considered similar to the original. While this may be feasible for transgenic manipulations involving genes whose products do not have core enzymatic functions (e.g. the bacterial *Cry* genes or viral coat protein gene), and to certain extent, when targeting secondary plant products like modification of flower color; core metabolism is often more difficult to manipulate because: a) it is homeostatically regulated, and b) it is highly webbed and interwoven with multiple other pathways. Consequently, changes in core metabolism have effects that are far reaching and may involve multiple pathways [1] and references therein, [2–4] and the references therein.

Two key aspects of studies aimed at understanding metabolic regulation in plants are: i) the ability to manipulate metabolism by using inhibitors, mutants or genetic engineering and genome editing, and ii) the ability to measure the impact of this change, i.e. the phenotype. Until the advent of microarrays, high throughput sequencing and metabolome analysis tools, the number of genes and metabolites that could be studied at any one time was rather limited. Thus it was imperative to decide *a priori* which genes and metabolites would be important to study. High throughput technologies have removed this bias by enabling global gene expression profiling, and to simultaneously analyze the pleiotropic effects of manipulating a metabolic pathway [5–12]. Furthermore the availability of new software platforms has enabled us to layer the outcomes of these diverse tools to develop connections between the two types of outcomes (i.e. transcriptomics and metabolomics). These techniques can reveal effects that are not only distal to the site of the manipulated step, but also may be unanticipated. What may on the one hand be considered a “fishing expedition” might more accurately be viewed as an entirely comprehensive systems study [13]. Therefore, it is possible that at some point in the near future, transcriptomic and metabolomic analyses of new genetically modified organisms will be a standard practice before their release into the field/market in order to identify inadvertent consequences of changes in gene expression and metabolism. While these techniques themselves have limitations (e.g. they do not measure changes in enzyme activities or metabolite fluxes), still they are valuable in detecting changes that may occur in branched pathways because few changes can happen in any branch of metabolism without concomitant changes in the expression of genes in related pathways. Metabolic profiling is a promising avenue to complement transcriptomics in global/systems analysis of metabolism [2, 4, 9, 14, 15].

Polyamines (PAs; putrescine – Put, spermidine - Spd, and spermine - Spm) are low molecular weight carbon (C) and nitrogen (N) rich compounds that are ubiquitous in living cells. Although many of their specific cellular functions in plants remain uncharacterized, they have been implicated in a variety of physiological responses and molecular interactions. The roles of PAs in plant growth and development, response to abiotic stress and Ca^2+^ deficiency, N sequestration, and their interactions with cellular macromolecules have been frequently discussed [16–23]. As sequences for key genes encoding PA biosynthetic enzymes have become available, metabolic perturbation (in particular up-regulation of specific steps) by genetic engineering of this pathway has become a routine strategy [24–31]. Since PA metabolism is part of a network of highly interdependent pathways, which are central to N metabolism and energy transformations (Additional file 1: Figure S1), it is hypothesized that altering PA metabolism will impact many of these and other pathways in the cells [32–36].

We have used two isogenic cell lines of poplar (*Populus nigra x maximowiczii*, clone NM6); one expresses a mouse Orn decarboxylase – m*ODC* cDNA and produces high Put (called HP), and the other is control (called GUS) that expresses a bacterial *β-glucuronidase* (*GUS*) gene. Both genes are under the control of a constitutive (2x35S CaMV – Cauliflower Mosaic Virus) promoter. The two cell lines were created at the same time [25, 31] and have been grown in vitro under identical (physical and chemical) growth conditions. By using isogenic cell lines we minimize the background variation between the control and the experimental (HP) cell lines that are being compared. Furthermore, the use of cell cultures simplifies complications related to the use of whole plants, e.g. heterogeneous tissue types and the translocation of metabolites between different organs. Building upon the information published earlier [25, 34–41], in the present study, we have investigated the broader impact of experimental manipulation of a single step in the PA metabolic pathway (i.e. the biosynthesis of Put from ornithine - Orn) on the transcriptome and the metabolome of these cells.

The two transgenic poplar cell lines (HP and the control) have been characterized over the years for various metabolic changes where the HP cells showed a consistently higher (3–to–10 fold) concentration of Put as compared to the control cells; Put content of the non-transgenic cells were always comparable to the control line. The HP cells also showed increased Put catabolism but without a change in the half-life of Put [25, 31]. Also, the native ADC (arginine decarboxylase) activity was not affected in HP cells [25]. The ACC (1-aminocyclopropane-1-carboxylate) and ethylene production were comparable in HP and the control cells, which suggested no competition between PA and ethylene biosynthetic pathways [37]. Although a small increase in Spd was seen in HP cells, neither the catabolism rate (half life) of Spd nor that of Spm was affected [31, 42]. Physiologically, there was greater plasma membrane permeability, increased amounts of soluble protein, enhanced tolerance to KNO_3_, and more susceptibility to NH_4_NO_3_ in the HP cells vs. control cells [38]. Increased PA catabolism in HP cells apparently led to accumulation of H_2_O_2_ accompanied by up-regulation of oxidative stress related enzymes (e.g. glutathione reductase and monodehydroascorbate reductase), leading to the conclusion that with millimolar quantities of Put there was a negative influence on the oxidative state of HP cells [34, 39, 40]. Certain anion transporters were affected in that in response to Al treatment, HP cells exhibited an apparent advantage over the control cells, which was explained by reduction in its uptake and increase in its extrusion [40]. Additionally, increases in the cellular contents of GABA, Ala, Thr, Val and Ile as well declines in several other amino acids (e.g. Glu, Gln, His, Arg, Ser, Gly, Phe, Trp, Asp, Lys, Leu, Cys, and Met, and already low Orn) were found in HP cells, with C and N assimilation being up-regulated concomitantly [34]. Increased utilization of Orn by mODC did not change the expression of genes in Glu-Orn-Arg pathway. It was postulated that apparently biochemical regulation controls this pathway rather than gene regulation [35, 43].

For the present study, using poplar microarrays, we have analyzed the transcriptomic data in three ways: (1) changes in expression of genes specifically involved in PA metabolism from N assimilation into Glu and beyond; (2) functional clustering, in order to examine the effects on specific areas of metabolism and cell functions; and (3) hierarchical clustering, in order to discover groups of genes that are potentially co-regulated in response to the enhanced PA metabolism. Likewise, the two cell lines have been compared for metabolite groups in pathways closely and distantly related to PAs and amino acids, and those pathways that constitute the core energy metabolism involving sugars, the organic acids and major N compounds. The results reveal transcriptomic as well as metabolomic changes that are widespread and go beyond the pathways related to PA metabolism, corroborating our earlier conclusions of pleiotropic effects of high Put production on amino acid metabolism and other physiological functions [34–36, 40].

## Methods

### Cell growth and harvest

The wild type suspension cultures of *Populus nigra x maximowiczii* (Clone NM6) were obtained from the Natural Resource Canada, Canadian Forest Service, Stn. Sainte-Foy, Quebec, Canada. The production and maintenance of HP and the control cell lines used here have been described previously [25, 31]. The former expresses a m*ODC* cDNA while the latter (that served as control) expresses the bacterial *GUS* gene; both cell lines also express the *neomycin phosphotransferase* (*NPT*II) selectable marker gene. All transgenes are constitutively expressed under the control of a modified 35S CaMV promoter. This strategy has allowed us to treat the two cell lines identically for maintenance of stocks during their culture history; e.g. growth in the presence of kanamycin. Liquid cultures were maintained on a weekly subculture routine and harvested for PA and other analyses as described [25, 31, 35, 36]. Over long term, solid cultures (callus) of the two lines were also maintained (on a monthly subculture routine), and used to restart the liquid cultures if they were lost (e.g. due to contamination). All cultures were grown in Murashige and Skoog [44] medium (solid or liquid) containing 3 % sucrose and 0.5 mg.L^−1^ 2,4-D (2,4-dichlorophenoxy-acetic acid) under 12 h light cycle (80–100 μE.m^−2^.sec^−1^) at 25 ± 1 C. The cells from liquid cultures were collected by vacuum filtration on Miracloth, washed quickly with de-ionized water, and weighed. For more details on cell storage and processing for various analyses, see Additional file 2: Supplemental Material.

### Experimental design for microarray analysis

The following comparisons in the transcriptomes of the two cell lines were made: (i) HP vs. the control (GUS) cells on day 3 and day 5 of the seven day culture cycle, and (ii) day 3 vs. day 5 of the culture cycle within each cell line. Consistency within and between microarrays was confirmed by making a number of quality control comparisons; significant changes in expression were evenly distributed without bias or large groups of outliers. Data were examined in a number of different ways as described below, all of which began with exclusion of data that failed coefficient of variation (CV) or dye swap tests.

Total RNA was extracted as described by Page and Minocha [45]. Following the removal of DNA (TURBO DNA-free^TM^ kit - Ambion Inc., Austin, TX) and quantification by NanoDrop (Thermo-Fisher, Wilmington, DE), mRNA was reverse transcribed using Superscript^TM^ Indirect cDNA Labeling System (Invitrogen, Carlsbad, CA). Procedures for labeling of cDNA with Cy3 and Cy5, microarray hybridization and washing of slides are described in Additional file 2: Supplemental Material.

Slides were scanned using a VersArray ChipReader™ scanner (BioRad, Hercules, CA) at 5 μm resolution with lasers set at 50 to 100 % so as to optimize the dynamic range and to equalize the signal from each channel [46, 47]. Detectors were set at 850 for all slides. Cy3 and Cy5 images were aligned and spots identified and quantified using VersArray Analyzer 5.0 (BioRad). Statistical analyses of the array data were performed after local background subtraction, omission of flagged spots, and conversion of data to a log base 2 scale using GeneGazer software (Bio-Rad). The procedure included LOWESS normalization using a smoothing parameter of 0.2 [32, 46]. Fluorescence intensity values were initially filtered by combining replicate experiments (average of mean ratios), and selecting spots with the quality CV values to <0.25. Spots having statistically significant differential expression for each comparison were identified using *t*-test with a false discovery rate p-value ≤ 0.05.

The widely accepted MIAME guidelines for microarray analysis and verification [48] (http://archive.fged.org/mged/Workgroups/MIAME/miame_2.0.html) were followed in addition to recommendations for the experimental design from other sources [46–49]. For each of the two-channel arrays, one channel was derived from cDNA of control cells and compared directly to cDNA derived from the HP cells. To allow for a more accurate detection of differentially expressed genes, three biological replicates were used with independent preparations of total RNA/cDNA from each cell line, collected on either day 3 or day 5 of the 7 d culture cycle. In addition, each time point included dye swap reciprocal two-color experiments for each biological replicate [46, 47, 50]. Thus, six data points per cDNA (three biological replicates each with two technical replicates) were used to ensure reliable statistical analysis of results.

The microarray slides (PICME poplar Pop_28K_3_1_4 arrays - http://www.picme.at) were checked using a dissecting microscope for uniformity of spots prior to pre-hybridization, which was performed for 30 min on a slow rotary shaker with just enough pre-hybridization solution (5X SSC, 0.1 % SDS, and 1 % BSA) to cover them. Slides were rinsed 20x in ddH_2_O in 50 mL conical tubes to avoid damage to spots. The slides were then placed in 95 C ddH_2_O for 1 min, washed in 100 % ice-cold ethanol for 15 s, dried by centrifugation (4 C, 2500 rpm, 2 min), and stored at 4 C. Hybridization was always performed within 1 h of pre-hybridization. Original data are available at: http://www.ncbi.nlm.nih.gov/geo/query/acc.cgi?acc=GSE79420.

A heat map was generated using R-project program (http://www.r-project.org/) with the microarray data. The specific Gene Ontology numbers associated with molecular function/biological process were obtained by searching with the gene names for Gene Ontology terms associated with plants using EMBL Quick go (http://www.ebi.ac.uk/QuickGO/). The ESTs (Expressed Sequence Tags) were accordingly classified and grouped based on the function associated with the genes for which they represent.

### Metabolomic profiling

Metabolome differences were studied on days 2, 4 and 6 of the 7 d culture cycle; these days were chosen to alternate with the transcriptome analysis over the 7-day culture cycle. Once again, comparisons were made within each cell line on different days and also between the control and the HP cells on the same days. In the metabolomic analyses, the spectra of all chromatogram peaks from each of the three independent biological replicates per treatment were compared with electron impact mass spectrum of authentic standards; peaks were then normalized to the internal standard and the fresh weight of each sample.

For derivatization prior to GC/MS analysis, the dried 80 % methanol extracts were treated with 80 μl of methoxyamine hydrochloride (20 mg.mL^−1^ in pyridine) at 40 C for 60 min, followed by the addition of 80 μl N-Methyl-N-(trimethylsilyl) trifluoroacetamide (Pierce Biotech., Rockford, IL) for 60 min at 65 C was used for the analysis. Gas chromatography was performed using a HP-5MS (30 m × 0.25 mm I.D. and 0.25 μm film thickness) capillary column with an Agilent 6890 N gas chromatograph coupled with 5973 MSD (Palo Alto, CA). The inlet and MS interface temperatures were kept at 250 C and the ion source temperature was adjusted to 230 C. Two microliters of the derivatized extracts were injected with a split ratio of 5:1 using He as a carrier gas kept at a constant flow rate of 1.3 mL min^−1^. The temperature program was an initial 5-min isothermal heating at 70 C, followed by an oven temperature increase of 5 C min^−1^ to 310 C, and final 10 min at 310 C. The temperature program was 5-min isothermal heating at 90 C, followed by an oven temperature increase of 5 C min^−1^ to 260 C for 10 min. The mass spectrometer was operated in positive electron impact mode (EI) at 69.9 eV ionization energy in m/z 50–800 scan range. The spectra of all chromatogram peaks were evaluated using the HP Chemstation (Agilent, Palo Alto, CA) and AMDIS (NIST, Gaithersburg, MD) programs. The spectra of all chromatogram peaks were compared with electron impact mass spectrum libraries NIST08 and WILEY08 (Palisade Corporation, NY), and two custom-built libraries. To allow comparisons between samples, all data were normalized to the internal standard (hentriacontanoic acid at 10 mg.mL^−1^ – Sigma, St. Louis, MO) in each chromatogram, and the fresh weight of each sample. The recovery of metabolites was evaluated by adding authentic standards, drawn as representatives of several compound classes, to the cell samples after the extraction, followed by comparison with the pure standard mixture at the same concentration.

### Analysis of metabolite and transcriptome profiles

Data sets containing 3 independent biological replicates per sample were statistically analyzed by ANOVA and *t*-test using the algorithm incorporated into MS Excel (Microsoft Corp., Seattle, WA). Differences were statistically significant at p ≤ 0.05. Multiple testing - Tukey's test (HSD) with 5 % risk was done with XLStat 2007 program (Addinsoft, New York, NY). To assess the metabolic changes or differences between samples and to identify metabolites involved in group discrimination, Principal Component Analysis (PCA) was performed on log-transformed (to avoid the highest intensity peaks dominating and to make the values closer to a Gaussian distribution), mean-centered data using SIMCA-P+ (12.0.0.0) program (www.umetrics.com/simca). Additional data are available at: https://mynotebook.labarchives.com/share/ulav72/MjIuMXwxNzEzMTkvMTcvVHJlZU5vZGUvMzg1Mzg2MTkxNHw1Ni4x.

## Results

### Cell lines and the experimental design

The two cell lines used here for comparison have been described earlier [25, 35, 36]; their important physiological and biochemical properties have been summarized above under Introduction. During the experimental period, the HP line accumulated 4–5-fold higher amounts of Put than the control line. In deference to increased Put in HP cells, the cellular content of Spd was affected only slightly, and no significant difference was seen in its Spm content. The experimental design for the current study included a comparison of transcriptomes and metabolomes of the control and the HP cells on different days of the seven-day culture cycle. Changes in gene expression and cellular metabolites with time of culture within each cell line were also examined.

### High putrescine production/accumulation is associated with major changes in the transcriptome

**Fig. 1:**
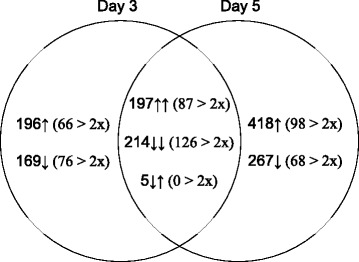
Venn diagram representing numbers of ESTs showing significant differences (p ≤ 0.05) in expression among the HP and the control cell lines on different days of analysis. Those, which are specific to day 3 or 5, are shown in individual circles and those that changed on both days are shown in overlapping circles. Arrows denote increase or decrease in expression in the HP cells relative to control cells; numbers in parentheses denote >2-fold change in expression. All data passed CV and dye swap tests

A summary of results on the ESTs that passed the various tests and those that were differentially expressed on days 3 and 5 in the two cell lines are shown in Fig. 1 and Additional file 1: Figure S2. Out of a total of 10470 and 16101 ESTs passing the quality control tests for days 3 and 5, respectively, about 7–8 % showed significant difference between the two cell lines on either day of analysis. When these ESTs were examined further, 214 out of 416 were found to show differences greater than two-fold on both days; they were split evenly between those that were up-regulated in the HP cells (197 ESTs, 87 > 2 fold) and those that were down regulated (214 ESTs, 126 > 2 fold). Of the 365 ESTs whose expression was different on day three only, 66 were up regulated and 76 were down regulated >2 fold; for others the difference was significant but <2-fold. The corresponding numbers of ESTs differing >2 fold between the two cell lines only on day five were 98 (up-regulated) and 68 (down-regulated), while the total number of genes showing significant difference was 418 (up regulated) and 267 (down regulated). There were at least 5 ESTs whose expression was significantly reduced in the HP cells on day three, and increased on day five but none greater than 2-fold.

#### Genes involved in polyamine metabolism

Gene models for all of the 27 genes presumably involved in PA metabolism from the first step of nitrogen (N) assimilation to the actual production of the three PAs (Additional file 1: Figure S1, Additional file 3: Table S1) were identified in the *Populus* genome. BLAST analysis at PICME (www.picme.at) of gene models (http://genome.jgi-psf.org/Poptr1/Poptr1.home.html) showed that ten of the genes in the PA and associated amino acids (Arg and Pro) pathway, namely AS, *CARB, DAO, NAGK, NAGPR, NAOD, NAOGAcT, ODC*, OTC, and *SPDS,* were in fact not represented on the microarray chip used in this study (Additional file 3: Table S1; for abbreviations, see Additional file 1: Figure S1). The remaining 17 genes were represented by a total of 106 ESTs, comprising 47 gene models. These were not distributed evenly; e.g. while a single spot each represented two genes (*AL* and *CARA*) others had greater redundancy. Most notable of the latter group was *Gln synthetase* (*GS*) with 28 spots representing seven gene models, and *SAMDC* with 15 spots representing four gene models. While small variations were seen in the expression of several of these genes, none varied more than two fold between the two cell lines on a given day or within the same cell line on different days.

#### Functional clustering of genes affected by high putrescine

The ESTs showing >2-fold difference in expression between the HP and the control cells on day three only, on day five only, and on both days were subjected to functional clustering analysis. Because a large proportion of the poplar genome sequence has not yet been functionally annotated, it is difficult at present to assign specific functional designations to many of the ESTs on the array. However, we used the Gene ontology terms (http://go.princeton.edu) for defining the function associated with the ESTs representing each gene. They are grouped into the following general functional categories: enzymatic function, transcription/translation (including ribosomal genes), membrane transport and osmoregulation, stress/wound induced, cell wall, unknown/unnamed (not annotated), and others (Additional file 3: Tables S2-S4).

Ten ESTs (7 genes) related to enzymatic function cluster varied between the two cell lines on both days (Additional file 3: Table S2), far less than those that varied on either day individually. All except two (glutathione S-transferase and an acetyl-transferase) were down regulated in the HP cells compared to the control cells both on days 3 and 5. On day 3 most of the genes associated with photosynthesis, glycolysis and methionine biosynthesis were down regulated (Additional file 3: Table S3). While genes related to ubiquitin cycle (5 ESTs) were down regulated, one EST related to polysaccharide catabolism was up regulated on day 3. Conversely, on day 5, one EST corresponding to ubiquitin cycle was up regulated (Additional file 3: Table S4). Overall more ESTs (11) were up regulated on day 5 compared to day 3.

Three of five ribosome/transcription/translation associated protein-encoding genes including histone 2A were up regulated on both day 3 and day 5. Among this group, seven genes were up regulated on day 3 while eleven were down regulated on day 5 (Additional file 3: Tables S3 and S4). In contrast, for membrane-associated genes, several were up regulated on day 5 alone. A plasma membrane intrinsic protein (8 ESTs) was found to be up regulated almost ten-fold on both days. An annexin (Anx1), an aquaporin TIP3 and an osmotin were down regulated on both days and the expression of an additional (unknown) aquaporin was higher on day 5. The transcripts of seven out of eleven genes in the group of stress/wound response related proteins were higher in HP cells. ESTs encoding chitinases and metallothioneins showed greater abundance while peroxidases and pathogenesis-related protein encoding ESTs were down regulated. All six of the cell wall associated protein genes (28 ESTs) identified in the microarrays were down regulated in the HP cells on both days; the most striking among this group were 25 ESTs in the extensin gene family.

Of the 35 gene models (represented by 45 ESTs) encoding proteins with known enzymatic functions (excluding those in the PA pathway), whose expression pattern was significantly different between the two cell lines on day 3 or day 5, a majority (24 gene models – 31 ESTs) were down regulated in the HP cells (Additional file 3: Tables S3, S4). Several genes in this group are involved in carbohydrate and phenolic metabolism, which may be responsible for metabolomic changes observed in the HP cells (see below). On the other hand, a larger number of genes associated with stress/wound response were up regulated in the HP cells on either day of analysis. Comparing differences within the same cell line on different days, fewer genes were up regulated in the HP cells on day 3 (17) vs. day 5 (24). In general ESTs encoding defense-related chitinases, heat-shock proteins, cold-stress proteins, and metallothioneins were up regulated in the HP cells; those related to peroxidation, cell wall maintenance, dehydration stress and carbohydrate metabolism were down regulated.

#### Hierarchical clustering

**Fig. 2:**
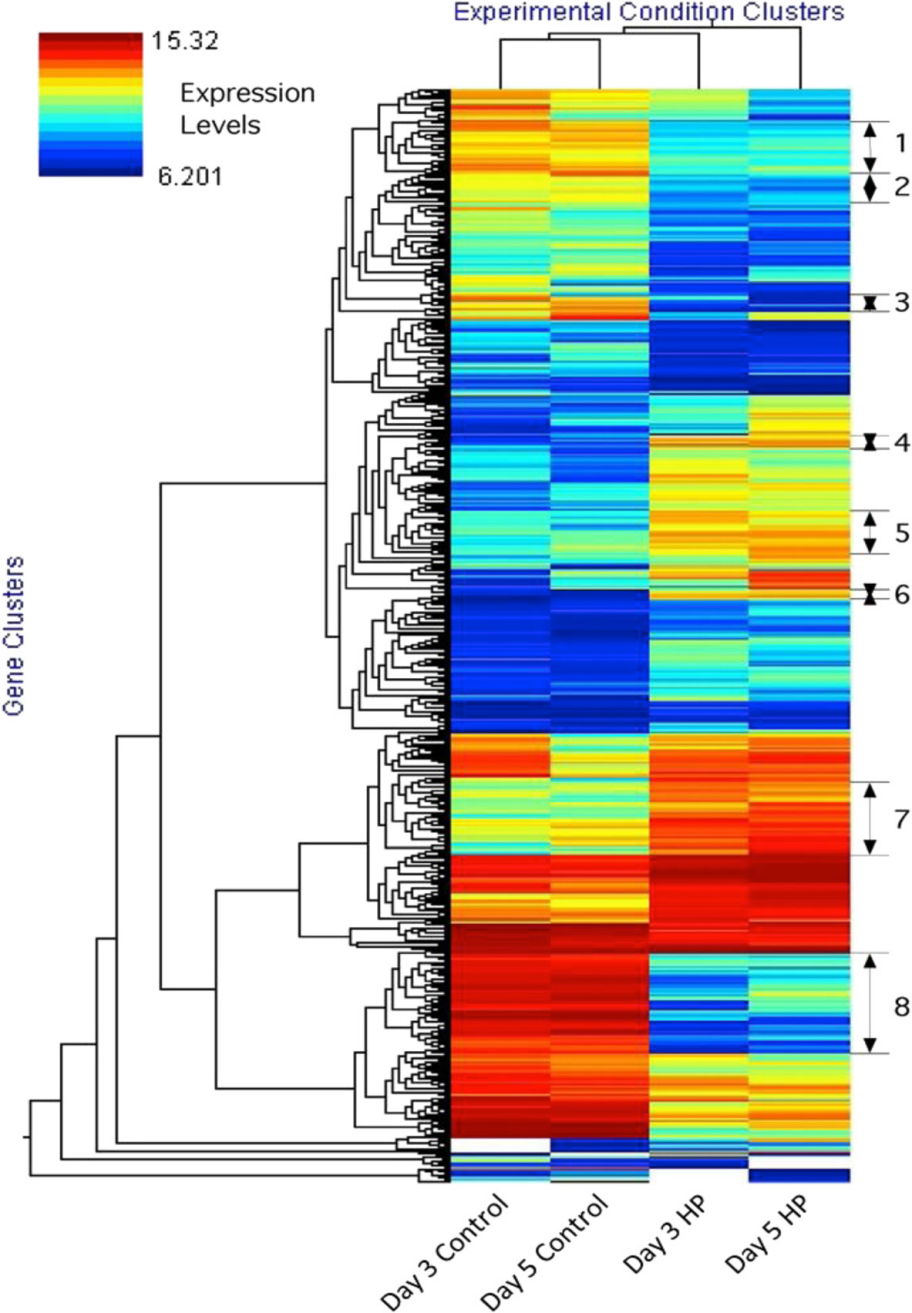
Hierarchical clustering of gene expression profiles in control and HP cells showing significant (p ≤ 0.05) differences between the two lines on different days of analysis. Heat map scale indicates spot intensities after local background subtraction. Blue indicates low-intensity spots while Red indicates spots with high signal. As expected, day 3 and day 5 controls appear similar to one another, as do the day 3 and day 5 HP cells. Transcripts were clustered into 8 distinct groups (1–8) based on co-regulation of gene expression patterns that were significantly up or down regulated on both day 3 and day 5 as compared to controls cells

Hierarchical clustering of genes with significant (p ≤ 0.05) differences between the control and the HP cells is shown in Fig. 2. As expected, the gene expression profiles of day 3 and day 5 control cells appear similar to one another, as do those for days 3 and 5 of HP cells. Some clusters can clearly be seen to be up or down regulated in response to Put manipulation. We used self-organizing maps to identify eight gene clusters for which expression is significantly up regulated for four (groups 4, 5, 6 and 7) and whose expression is down regulated in HP cells on both day 3 and day 5 vs. the control cells for four (1, 2, 3 and 8). The clusters contain genes that show a similar expression pattern but not necessarily belong to closely related pathways. The most obvious of these clusters is the strong down-regulation of extensin and PVR3 type genes in the HP cells (Group 8). Overall, the expression profiles of the HP cells were more similar to each other on the two days of analysis than those of the control cells.

### Metabolomic changes in relation to high putrescine production/accumulation go beyond the polyamines and amino acids

Analysis of metabolites by GC/MS detected a total of 645 compounds in the control cell line and 680 in the HP cell line. Of these, 190 and 178 compounds were positively identified in the control and the HP cells, respectively (Additional file 3: Table S5); the total number of positively identified compounds being >200. Fifty two percent of these metabolites were significantly altered (p ≤ 0.05) on different days during the seven-day culture period in the control line and 37 % in the HP cell line (Additional file 3: Table S5 – bold font). Comparing the two cell lines over the entire culture period revealed that >30 % of the total identified metabolites were present at higher concentration in the HP cells vs. the control cells; the HP cells had only 13 % of the compounds that were lower in concentration than the control cells.

**Fig. 3:**
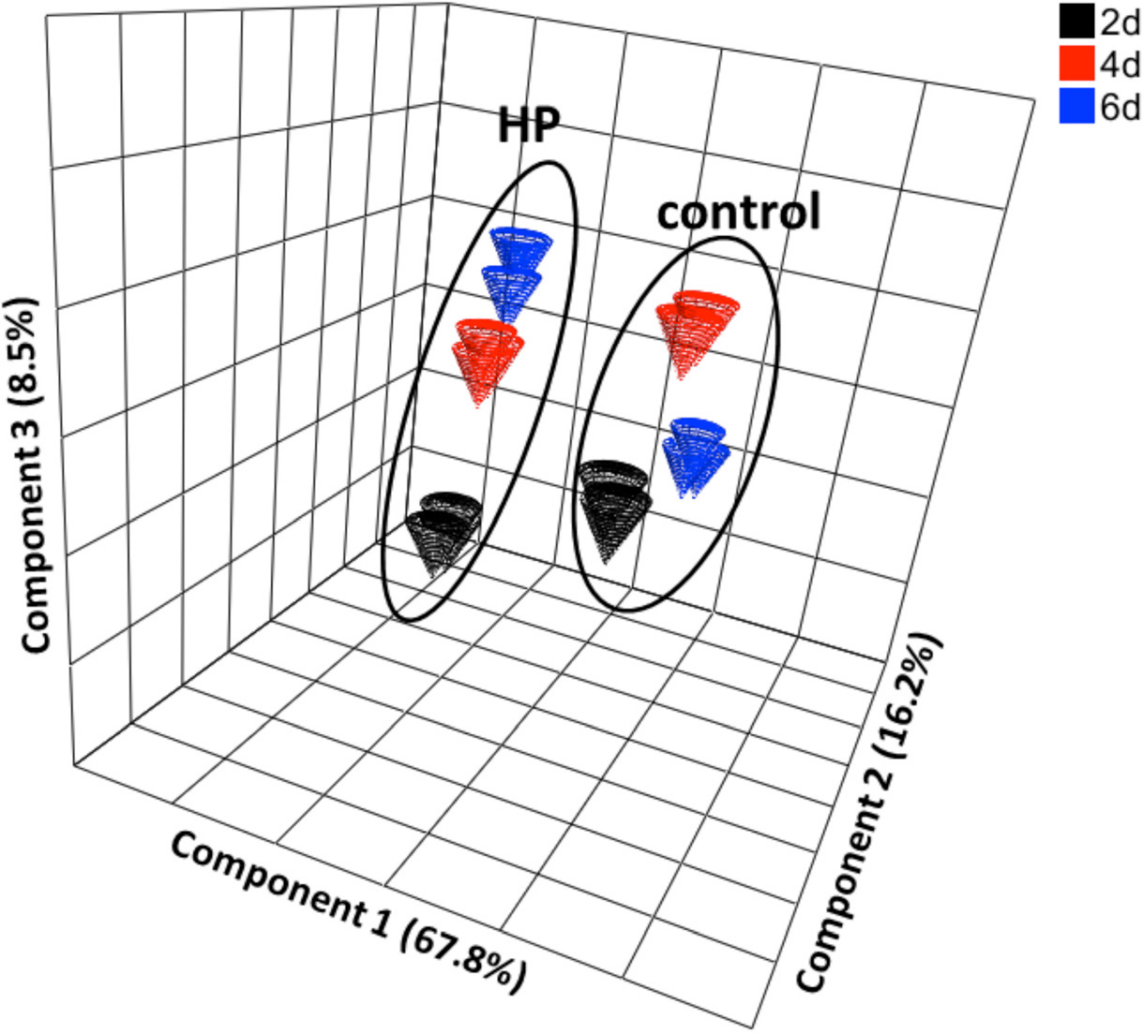
Principal Component Analysis (PCA) plot derived from the GC/MS spectra of extracts obtained from control and the HP cells on days 2 (black), 4 (red), and 6 (blue). Variability analysis described by two major components can be ascribed largely to differences in cell lines (PCA1 = 67.8 %) and to a lesser extent to the day of culture (PCA2 = 16.2 %)

When relative concentrations of the identified compounds (~200) were compared between the control and the HP lines on the same day of culture, about 60 % of them were found significantly altered (P ≤ 0.05) in HP cells on days 2, 4 and 6. Still the differences between the cell lines were far more dominant as compared to those within the same cell line on different days of analysis (Fig. 3 and Additional file 1: Figure S3A). On day 2 of culture, 27 % of the total identified metabolites were higher in the HP cells (vs. the control cells) compared to 36 %, which were lower in these cells (P ≤ 0.05). Of about 70 metabolites that differed more than two fold on all three days of analysis, the major groups were amino acids, organic acids, and a few carbohydrates; about half were up regulated and half were down regulated. Several of the amino acids that were up regulated belonged downstream of the PA-related pathways (e.g. GABA and Ala), and those that were down regulated were from the substrate side of the pathway; e.g. Glu, Gln, Acetyl-Glu, (Table 1). Among sugars, some of the hexoses and hexose-phosphates were up regulated in HP cells at all-time points; as were several amines (including Put, Cad, ethanolamine) and many organic acids. Several amino acids, most of the TCA cycle intermediates, and disaccharides (and their phosphates) were down regulated (Additional file 3: Table S1 and S5). Respective changes in 4 d old cultures included increases in 26 % and decreases in 33 % of the metabolites (calculated from data in Additional file 3: Table S5), and on day 6, these numbers were 26 % (increased) and 30 % (decreased). Both 4 and 6 day old cells showed the same trends of changes in the classes of the metabolites that changed (Table 1).

**Table 1:** Metabolites that changed significantly (p ≤ 0.05) on at least two different days of analysis during the seven-day culture cycle in the HP vs. the control cells. Data derived from Additional file 3: Table S5. More details are avilable at: https://mynotebook.labarchives.com/share/ulav72/MjIuMXwxNzEzMTkvMTcvVHJlZU5vZGUvMzg1Mzg2MTkxNHw1Ni4x

Up 2, 4 and 6 day	Down 2, 4 and 6 day	Up 2 and 4 or 4 and 6 or 2 and 6 day	Down 2 and 4 or 4 and 6 or 2 and 6 day
1,3-Dexadecanoylglycerol	Tritriacontanol	Gluconic acid	Hexadecanoic acid
Adenosine	Hexadecanoic acid, 1-[[oxy]methyl]-1,2-ethanediyl ester	Pyruvic acid	Inositol-P
Alanine	Eicosanoic acid	Serine	9,12-Octadecadienoic acid
B-alanine	Pentadecanoic acid	Ribose	Guanine
Amino-isobutyric acid	Tetracosanoic acid	2-Methylserine	Glucose-6-phosphate
Cadaverine	Triacontanoic acid	2-Aminobutyric acid	Pyroglutamic acid
Ethanolamine	Butylamine	2-Aminoethyl-phosphate	Methylthioadenosine
GABA	Glutamic acid	9-Octadecenoic acid	Homocysteine
Galactosamine	Glutamine	Pipecolic acid	Lysine
Glucosamine	Histidine	2-O-Glycerol-α-D-galactopyranoside	
Hydroxylamine	Inosine	Glucaric acid	
Putrescine	Leucine		
Suberylglycine	Methionine		
Uracil	N-Acetyl-glucosylamine		
Urea	N-Acetylglutamate		
Galactopyranose	N-Acetyl-Lysine		
1-Aminocyclopropane-carboxylic acid	Phenylalanine		
Arabinoic acid lactone	Threonine		
Galactaric acid	Tyrosine		
Galactonic acid	Aconitic acid		
Hydroxymalonic caid	Citric acid		
2-Indolecarboxylic acid	3-Hydroxymethyl-glutaric acid		
Malonic acid	2-Keto-gluconic acid		
Succinic acid	Lactic acid		
Digaactosylglycerol	Malic acid		
Fructose	Quinolinic aci		
Galactofuranose	Shikimic acid		
Galactopyranose	Xylonic acid, lactone		
Galactose	1,6-Anhydroglucose		
Glucose	Fructofuranoside		
1-Methyl-α-D-galactopyranoside	Gentiobiose		

**Fig. 4:**
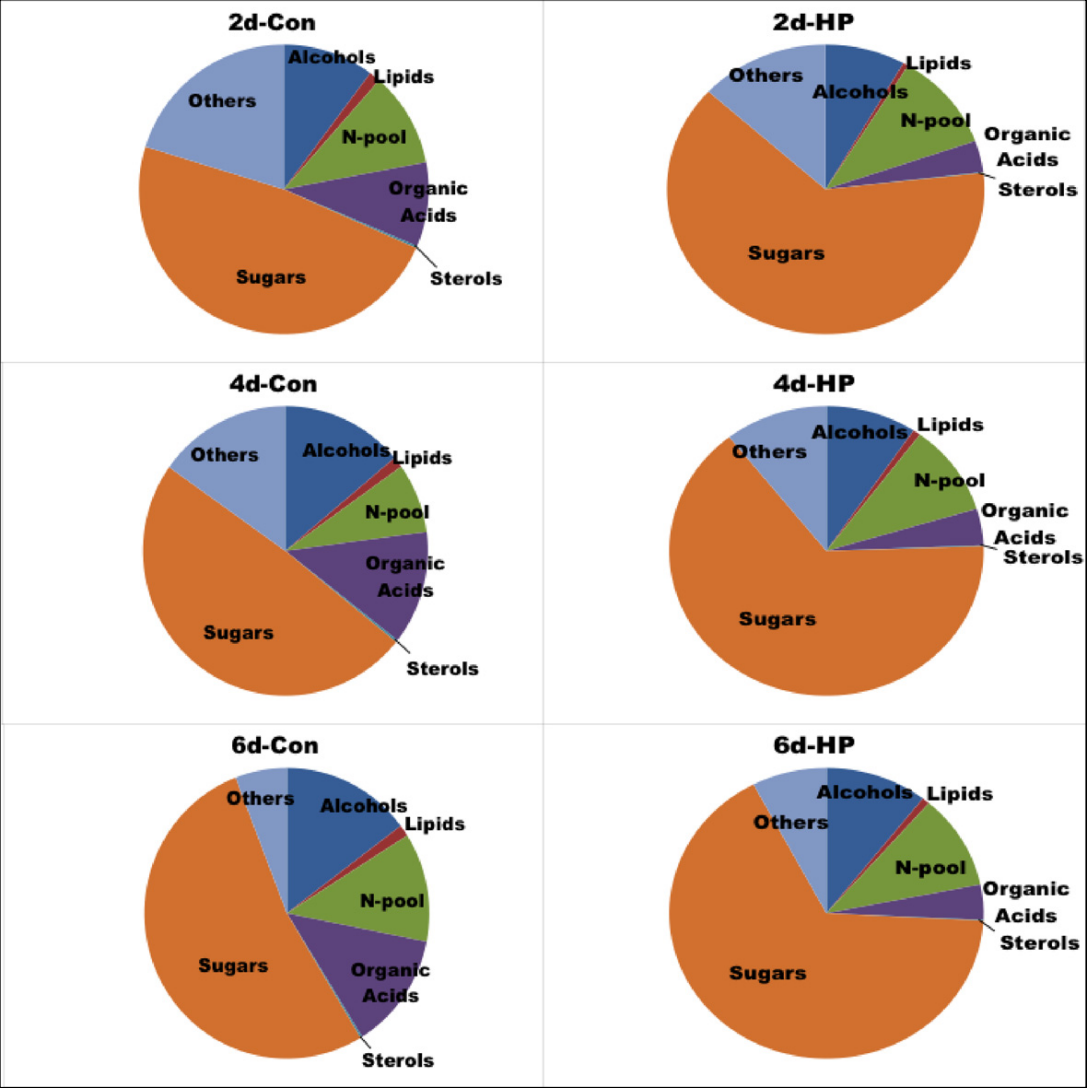
Relative composition (% of total) of major metabolite groups, and how they changed on different days of culture (2,4, and 6 d) for control and HP cells. Only those metabolites that were positively identified are included here (detailed in Additional file 3: Table S5)

The grouping of known metabolites that differed between the two cell lines and how they changed with time of culture within a cell line showed that the largest differences were seen for sugars followed by organic acids, nitrogenous compounds, and alcohols in that order on all three days of analysis (Fig. 4). The relative pools of sugars were higher in the HP cells on all days of comparison. Major (>2–5 fold) increases (in order of their relative abundance) were seen in glucose, fructose, galactopyranose, sorbose, sorbopyranose, glucopyranose, and galactose; sucrose (which was supplied in the growth medium) was the only major sugar that was lower in the HP vs. the control cells (Additional file 3: Table S5). Among the major organic acids that declined in the HP cells were malic acid, 3-hydroxymethylglutaric acid, citric acid, and quinolinic acid; succinic acid (a catabolic product of Put) being the only major organic acid whose content was higher in the HP cells. The nitrogenous compounds whose cellular concentrations increased (besides putrescine) included Ala, ethanolamine and GABA, while Glu and Thr were higher in the control cells. Less abundant nitrogenous compounds that increased >3 fold were Cad, β-Ala, and adenosine; several other low abundance amino acids had lower concentrations in the HP cells.

The metabolite changes in the HP cells represented several metabolic pathways; prominent among them were amino acid (N) metabolism, carbohydrate metabolism and lipid metabolism. The eleven dominant metabolites (relative concentration ≥1000 units – Additional file 3: Table S5) in both cell lines at most times were inositol, phosphate, monomethylphosphate, fructose, sorbose, sorbopyranose, galactose, galactopyranose, glucose, glucopyranose, and sucrose; however, their relative abundance was different in the two cell lines. For example, the relative concentrations of fructose, glucose, sorbose and galactopyranose were much lower than sucrose in the control cells; in the HP cells, these were among the dominant metabolites along with galactose, glucopyranose, sorbopyranose and Ala. While differences (between HP and control cells) in the concentrations of inositol, sucrose and phosphate were <2.0 fold, for other dominant metabolites, the differences were >3–4 fold. Additional compounds found in relatively large concentrations (>100 units) that were >2-fold higher in concentration in the HP cells on all days were ethanolamine, GABA and succinic acid, and of course Put; those that were >2-fold lower included glutamic acid, citric acid, malic acid, quinolinic acid, 2-ketogluconic acid, hydroxymethylglutaric acid, and fructofuranoside.

When the ordination scores from Principal Component Analysis were subjected to analysis of variance to determine the major sources of variation between the samples, they were mostly distinguished based on cell line specificity (control vs. HP - Fig. 3; also see Additional file 1: Figure S3B-D) on the first principal component (67.8 % of explained variation); their time points discriminated on the second PC (16.2 % of total variability). In order to identify the sources of variation between control and the HP cells during the culture period, each pair (control 2d-HP 2d, control 4d-HP 4d and control 6d-HP 6d) was subjected to additional PCA that revealed clear differences between the HP and the control lines on all three days of analysis (Additional file 1: Figure S3B-D). The contribution of individual metabolites to the discrimination of control and HP lines showed that high Put production significantly altered many metabolites related to carbohydrate and N metabolism, in addition to those involved in organic acid, lipid and alcohol metabolism.

## Discussion

Before completion of the draft of poplar genome sequence [51], several microarray studies in poplar had been published, which involved analysis of gene expression during development of tension wood [52], effects of CO_2_ and ozone [53, 54], effect of safener [55], cambial dormancy and growth [56, 57], cell death [58], and herbivory [59]. The subject areas investigated since then include disease [60] and drought [61, 62] responses, growth and hormonal regulation [63, 64], salt and osmotic stress [65, 66], and carbon monoxide response [67]. Janz *et al.* [65] compared changes in the metabolome and the transcriptome of a tolerant (*P. euphratica*) and a sensitive (*P. x canescens*) species of poplar in response to salinity. Whereas analysis of transcriptomes showed no broad activation of the ‘general’ stress response genes, the salt tolerant species did show several metabolic changes, particularly in primary sugars, sugar alcohols, and phenolics as compared to the salt-sensitive species. The metabolomic changes were accompanied by alterations in the expression of several genes involved in these pathways.

Here we report a comprehensive transcriptomic and metabolomic analysis of the effects of genetic manipulation of a single metabolic step to achieve higher accumulation of a biologically active metabolite (i.e. Put), which plays a wide range of physiological roles in plants. In contrast to previous transcriptomic and metabolomic studies in poplar that used whole plants (which exhibit high degrees of variability between biological replicates resulting in a large proportion of genes/metabolites being eliminated from analysis), the present study used *in vitro*-grown cell cultures. Most studies with animals as well as plants have relied upon the premise that regulatory aspects of metabolism in cell cultures reflect the actual situation in the whole organism. Being isogenic in origin and homogeneous in growth habit, the cell cultures show far less inherent variability among experimental replicates, thus providing greater sensitivity to detect changes in gene expression and metabolic profiles due to the expression of a transgene.

### High putrescine biosynthesis/accumulation has pleiotropic effects on the transcriptome and the metabolome

The observed differences in transcription of a diverse group of genes and the metabolites belonging to multiple pathways clearly augment the previously reported pleiotropic physiological, biochemical and molecular responses of cells to elevated PA contents [20, 21, 33–36, 39, 40]. A similar study of genetic manipulation of PA content of transgenic tomato fruit using yeast *SAMDC* was also shown to cause major and apparently unrelated metabolic and phenotypic changes including increase in lycopene, prolonged vine life, and enhanced fruit juice quality [68–70]. It is further evident that metabolic changes seen in the HP cells reflect the close interaction between N and C metabolism in that major changes are seen in sugars, organic acids, and stress-related compounds, which are directly related to N metabolism. Other groups of metabolites like alcohols, lipids and phenolic compounds are less affected by metabolic engineering of the PA pathway.

Our functional clustering of the transcribed genes reveals broader impact of high Put production on gross cell physiology, including diverse processes like osmotic regulation, cell wall biosynthesis, and transcription and translation. We also discovered several groups of genes whose expression changed in similar ways in the HP cells, demonstrating areas of cellular activity or metabolism that exhibit a coordinated response to experimental manipulation of Put. Although the lack of extensive genome annotation hinders identification of many of these genes, it can be argued that changes in their expression are related to the observed grouping of pathways associated with the metabolism of sugars, organic acids and N-rich compounds, as revealed by the metabolome analysis. A direct correlation between changes in the expression of specific genes/enzymes with the metabolites that are altered must await completion of the ongoing gene identification efforts in poplar. Contrary to the expectation that up regulation of the ODC step (Orn → Put) might first cause adjustments in the remaining parts of the PA pathway (e.g. down regulation of the ADC step and/or up regulation of Orn biosynthetic genes), only small and insignificant differences were seen in the expression of the Glu → Orn → Arg → Put pathway genes. The lack of major differences in the expression of genes in this component of the pathway supports the earlier argument that the metabolic regulation of this pathway occurs mostly at the levels of enzyme activity and substrate availability [35, 71]. In Page et al. [35], we reported earlier that most of the PA biosynthetic genes show coordinated expression, falling into two distinct clusters which exhibit parallel changes in expression with the time of culture in the growth medium.

Ornithine occupies a crucial position in the metabolic pathway of Arg biosynthesis from Glu in plants (Additional file 1: Figure S1), and is itself present in the cells in very low concentrations (40 to 50 fold less than either Glu or Arg) [34, 71]. Since the HP cells produce several-fold higher amounts of Put from Orn, it can be hypothesized that the production of Orn from Glu must be enhanced enormously to keep pace with its consumption. This involves a series of at least six enzymatic steps; the presumed regulatory step being the commitment of Glu to the Orn pathway through its acetylation by NAGS [71–74]. Contrary to the expectation that expression of these genes may be up regulated to meet the demand for increased flux of metabolites from Glu → Orn → Arg, none of these genes showed a significant change in expression (based on several ESTs in most cases) in response to increased production of Put and enhanced utilization and synthesis of Orn in the HP cells. These results are consistent with the RT-qPCR analysis data published earlier [35, 36], and provide an internal control for verification of the microarray protocols used here. The observed reductions in precursors of this pathway (i.e. Glu and Gln) are supportive of the argument that the entire pathway from Glu to Orn is hyper-activated by the increased consumption of Orn [33, 71]. At the same time, increases in GABA, succinate and β-alanine corroborate increased catabolism of PAs as demonstrated directly in our earlier studies [31]. These results are analogous to the observations of little or no change in the transcription of any of the genes coding for enzymes involved in Tyr biosynthesis when its consumption was enhanced several fold by metabolic engineering of dhurrin in *Arabidopsis* [14].

The differences in metabolic profiles of the two lines were dramatic in magnitude and diverse in groupings in that on any given day of analysis, more than 60 % of the metabolites were significantly different between the two cell lines; about 25 % of them were higher in the HP cells and 36 % were lower (Additional file 3: Table S5). The PCA of metabolic changes with time-of-culture within a cell line and those between the two cell lines on the same day of culture provides ample evidence that the observed changes are related to (if not caused by) the genetic manipulation of Put, and not merely a manifestation of differences due to nutrients in the medium on different days of culture. However, it should also be emphasized that enhanced production of Put itself has a multitude of other physiological effects, particularly related to oxidative metabolism (see above under Introduction); therefore, it is arguable that some of the observed metabolic changes are indirect effects of high Put accumulation and its catabolism.

Major metabolite changes in HP cells like reductions in the contents of almost all amino acids (except GABA, Ala and Ser, all of which are catabolic products of PAs) are consistent with increased utilization of Glu/Gln for higher amounts of Orn production. Metabolomic analysis did not show noticeable changes in most of the other intermediates in the Glu → Orn → Arg pathway, indicating either that these metabolites are all present transiently, and in low quantities (i.e. not accumulated) or the technique did not allow for their detection and identification. Unfortunately, Arg, Orn, and citrulline could not be analyzed separately with the metabolite profiling technique due to their uncontrolled conversion to Orn during the trimethylsilylation process [75]. Mohapatra *et al.* [34] have earlier shown by HPLC analysis of amino acids that while Orn concentration is substantially lower in the HP cells, the concentrations of Arg are quite similar in the two cell lines.

### Changes in cellular metabolism in HP cells are consistent with physiological effects of PAs

Polyamines have been implicated in a wide variety of physiological and biochemical processes, especially in plant growth and development, response to abiotic stress and Ca^2+^ deficiency, N sequestration, and interactions with cellular macromolecules [21, 23, 76, 77]. The changes in gene expression observed in HP cells therefore could be related to either the cellular response to accumulation of high Put or a secondary response dealing with amelioration of the harmful effects of high Put; e.g. those associated with increased oxidative stress [39].

The fact that there were no ribosomal genes that showed significant change in expression between the two cell lines on either day of analysis, while several differed on different days of culture within the same line, perhaps is a reflection of the changing rates of total protein biosynthesis in relation to the time of culture and nutrient availability. The increase in expression of several ribosomal genes in HP cells on day 3 is in agreement with the observation that they contain higher soluble protein content during the first 2 to 4 days of culture on transfer to fresh medium [35, 36]. The HP cells do not maintain higher protein content during the latter part of the 7 d culture cycle, and enter a phase of decline in protein content sooner than the control cells. This is corroborated by the observation that on day 5, eight different ribosomal gene models showed lower expression in the HP cells.

A major surprise was the observed reduction in the expression of several cell wall related genes in the HP cells vs. the control cells; none showed higher expression. The greatest contributors to this category were the extensins, whose expression was reduced in the HP cells to an average of 10 % of that seen in the control line. Also among the down-regulated cell wall associated genes in the HP cells were 3,5-epimerase/4-reductase (involved in cell wall sugar metabolism), a fasciclin like AGP 10 (involved in cell adhesion), and a cell wall-plasma membrane linker protein. While there is no obvious explanation for these changes, they may reflect the observed differences in a broad spectrum of carbohydrates in the two cell lines, which are involved in the biosynthesis of cell wall. Polyamines have been shown to play significant role in the cell wall metabolism specifically in relation to lignin deposition [20, 21].

Gene models associated with shock, stress or wound response showed some similarity with genes involved in membrane transport and osmoregulation (functions commonly associated with PA effects) which were predominantly down-regulated on day 3 and up-regulated on day 5. Genes in this category, which showed significant differences in expression between control and HP cells on both days, fell into two groups. The first included chitinase, chitin binding- (hevein), and wound-induced and heat-shock proteins; these proteins are associated with cell wall degradation and in defense responses, and were up regulated in the HP cells. The second group included ascorbate peroxidase, a cationic peroxidase, and a “stress related” gene, which showed reduced expression in the HP cells; they perhaps reflect observed changes in the oxidative state of HP cells in relation to high Put [39].

Other enzymatic genes showing changes in expression included a diverse group of proteins. Two genes whose expression was reduced in the HP cells on day 3 or both days 3 and 5, encode two separate Met synthases; this is consistent with the lower Met content in the HP cells [40]. Likewise, the acetyl transferase, whose expression is up-regulated in the HP cells may be the one involved in the biosynthesis of Orn from Glu, the sub-pathway that must be up-regulated to meet the increased demand due to higher ODC activity in the HP cells [73, 74].

In a study like this where the transgenic cell lines have been maintained for several years (or for that matter where any mutant or transgenic lines of plants have been grown for several generations), it would be expected that the new metabolic homeostasis would lead to secondary metabolic effects that may be unrelated to the primary target of manipulation. Often, the primary cell line or the transgenic plants (e.g. T1 generation) generally do not provide sufficient plant material to do extensive experimentation. This is especially true when the transgene is constitutively expressed. Whether it is cell cultures or it is plants (seedlings or mature), the primary transformants have adjusted to the constitutive expression. Thus some of the results presented here are subject to interpretation of the long term homeostatic adjustments of the cells. More recently, working with *Arabidopsis*, we have generated constitutive as well as inducible (by estradiol) transgenic lines and investigated some of the similar biochemical aspects as with poplar cells, and found them to show similar results [33, 71].

## Conclusions

The key finding of physiological importance in this study is that up-regulation of a single metabolite through overexpression of a transgene may cause changes in the expression of a broad spectrum of genes and a multitude of metabolites which participate in diverse physiological functions; these results are consistent with the multifaceted functions of PAs in plants. The results presented here highlight the complexity of cellular responses to perturbation of a single metabolite in a rather simple group of undifferentiated cells. Combined with recent studies from our lab [33, 71] showing that (i) increased Put production causes an increased flux of Glu into Orn, with little concurrent effects on Pro and Arg (two other amino acids of the Glu-Orn-Pro-Arg-GABA pathway of N metabolism), and (ii) the increased use of Glu into this pathway is at least partially compensated by its increased production via additional N and C assimilation; the results may be useful in designing transgenic plants with increased N use efficiency as well as enhanced C assimilation, especially in plants intended for non-food/feed applications (e.g. increased biomass production or for biofuels).

### Availability of data and materials

The cell lines described here are being maintained at the University of New Hampshire in the laboratory of the Corresponding author (sminocha@unh.edu). Additional microarray data are available at: http://www.ncbi.nlm.nih.gov/geo/query/acc.cgi?acc=GSE79420 (contact Dr. L.C. - csekel@uah.edu); and additional metabolomics data are available at: https://mynotebook.labarchives.com/share/ulav72/MjIuMXwxNzEzMTkvMTcvVHJlZU5vZGUvMzg1Mzg2MTkxNHw1Ni4x (Contact A.U., ulanov@illinois.edu or ZL lucasli@illinois.edu).

### Ethics and consent to participate

Not applicable

### Consent to publish

Not applicable

## Additional files

Additional file 1: Figure S1. The pathway for the biosynthesis of polyamines and related metabolites starting from the assimilation of nitrogen (Adapted from Majumdar et al. 2016). Figure S2. (A, B) - Quality control scatter plots showing expression level for data that passed CV and dye-swap tests. Red spots indicate data that passed statistical analysis for differential expression between the control and the HP cells. Figure S3. The loading plots (S-plot) of the OPLS-DA results for the control and HP cell extracts on days 2 (A), 4 (B), and 6 (C). In the S-plot, each point represents a single metabolite (marker). The *x*-axis shows the variable contributions. The farther away a data point is from the 0 value, the more it contributes to sample variance. The *y*-axis shows the sample correlations within the same sample group. The farther away a metabolite is from the 0 value, the better is its correlation from injection to injection. As a result, the metabolites on both ends of the S-shaped curve represent the leading contributing ions from each sample group. The OPLS-DA is a multivariate analysis model which separates the systematic variation in X into two parts, one that is linearly related (and therefore predictive) to Y and one that is orthogonal to Y (unrelated); the Y-predictive/related part represents the between-class variation, the Y-orthogonal (ToPo) part constitutes the within-class variation. (DOCX 1653 kb) Additional file 2: Methods for RNA extraction, cDNA preparation and labeling, microarray hybridization and processing, and metabolomic analysis. Supplemental data Microarrays: http://www.ncbi.nlm.nih.gov/geo/query/acc.cgi?acc=GSE79420. Metabolomic Data: https://mynotebook.labarchives.com/share/ulav72/MjIuMXwxNzEzMTkvMTcvVHJlZU5vZGUvMzg1Mzg2MTkxNHw1Ni4x. (PDF 451 kb) Additional file 3: Table S1. Summary of changes in expression of genes involved in polyamine metabolism. For abbreviations see Fig. 1. Sequences for AS, CARB, DAO, NAGK, NAGPR, NAOD, NAOGAcT, ODC, OTC and SPDS were not represented on the microarray. Table S2. Functional clustering of gene models showing significant (P ≤ 0.05) differences (≥2 fold) between the HP and the control cell lines on both day 3 and day 5. Table S3. Functional clustering of gene models showing significant (P ≤ 0.05) differences (≥2 fold) between the HP and the control cell lines on day 3 only. Table S4. Functional clustering of gene models showing significant (P ≤ 0.05) differences (≥2 fold) between the HP and the control cell lines on day 5 only. Table S5. List of metabolites that were positively identified in poplar control and HP cell lines. ND = not detectable. Values that are significantly different (P ≤ 0.05) in the HP cells from the corresponding control cells on a given day are marked in bold. (DOCX 103 kb)

## Abbreviations

ACC1: Aminocyclopropane-1-carboxylate
ADC: Arginine decarboxylase
CV: coefficient of variation
2,4-D: 2,4-Dichlorophenoxyacetic acid
EST: expressed sequence tag
GABA: Gamma-aminobutyric acid
GUS: β-Glucuronidase
HP: high putrescine
NPTII: *Neomycin phosphotransferase*
ODC: Ornithine decarboxylase
PA: Polyamine
PCA: principal component analysis
Put: Putrescine
Spd: Spermidine
Spm: Spermine
SAMDC: S-Adenosylmethionine decarboxylase
