# Supplementary material for: Genetic manipulation of putrescine biosynthesis reprograms the cellular transcriptome and the metabolome
Source: BMC Plant Biol. 2016 May 18;16:113. doi: 10.1186/s12870-016-0796-2 (PMC4870780; doi:10.1186/s12870-016-0796-2)
Supplement: Additional file 1: Figure S1. — The pathway for the biosynthesis of polyamines and related metabolites starting from the assimilation of nitrogen (Adapted from Majumdar et al. 2016). Figure S2. (A, B) - Quality control scatter plots showing expression level for data that passed CV and dye-swap tests. Red spots indicate data that passed statistical analysis for differential expression between the control and the HP cells. Figure S3. The loading plots (S-plot) of the OPLS-DA results for the control and HP cell extracts on days 2 (A), 4 (B), and 6 (C). In the S-plot, each point represents a single metabolite (marker). The x-axis shows the variable contributions. The farther away a data point is from the 0 value, the more it contributes to sample variance. The y-axis shows the sample correlations within the same sample group. The farther away a metabolite is from the 0 value, the better is its correlation from injection to injection. As a result, the metabolites on both ends of the S-shaped curve represent the leading contributing ions from each sample group. The OPLS-DA is a multivariate analysis model which separates the systematic variation in X into two parts, one that is linearly related (and therefore predictive) to Y and one that is orthogonal to Y (unrelated); the Y-predictive/related part represents the between-class variation, the Y-orthogonal (ToPo) part constitutes the within-class variation. (DOCX 1653 kb) [file 12870_2016_796_MOESM1_ESM.docx]

## **Authors: Page A.F. et al.**

## **Additional file 1: Figures S1-S3**

**Additional file 1: Figure S1.** The pathway for the biosynthesis of polyamines and related metabolites starting from the assimilated nitrogen. ACC, 1-Aminocyclopropane-1-carboxylic acid oxidase; ADC, Arginine decarboxylase; AL, Argininosuccinate lyase; ARG, arginase; AS, Argininosuccinate synthase; CARA. Carbamoylphosphate synthase large subunit; CARB, small subunit of carbamoylphosphate synthase; DAO, Diamine oxidase; GAD, Glutamate decarboxylase; GOGAT, Glutamate synthase; GS, Glutamine synthetase; LysDC, Lysine decarboxylase; NAGK, N-Acetylglutamate kinase; NAGPR, N-Acetylglutamate-5-P reductase; NAGS, N-acetyl-glutamate synthase; NAOAT, N2-acetyl-Orn aminotransferase; NAOD, N2-acetyl-Orn deacetylase; NIR, Nitrite reductase; NR, Nitrate reductase; OAT, Ornithine aminotransferase; ODC, Ornithine decarboxylase; OTC, Ornithine transcarbamylase; SAMDC, S-adenosylmethionine decarboxylase; SPDS, spermidine synthase; SMPS, spermine synthase. Native ODC (heavier arrow) is not expressed in control cells, but the transgenic mODC is over-expressed in HP cells. (Adapted from Majumdar et al. 2016).


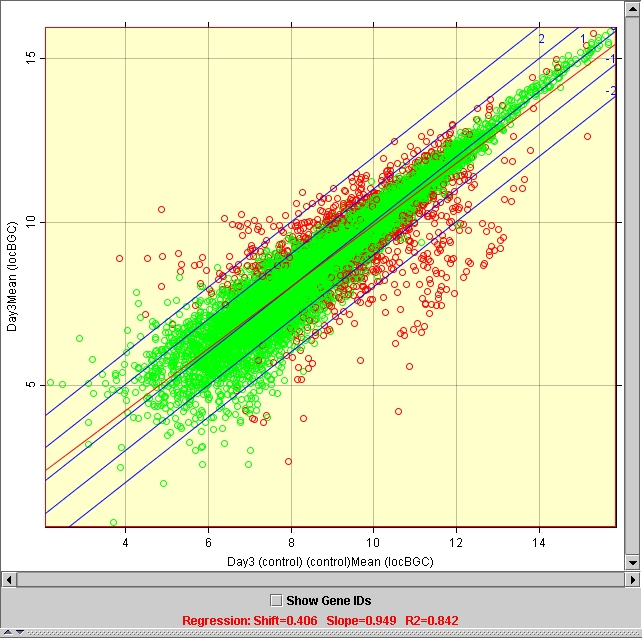


**Additional file 1: Figure S2.** Quality control scatter plots showing expression levels of data that passed CV and dye-swap tests. Red spots indicate data that passed statistical analysis for differential expression between control and HP cell lines. For original data, see: <http://www.ncbi.nlm.nih.gov/geo/query/acc.cgi?acc=GSE79420>.


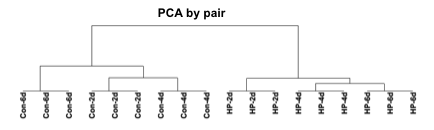

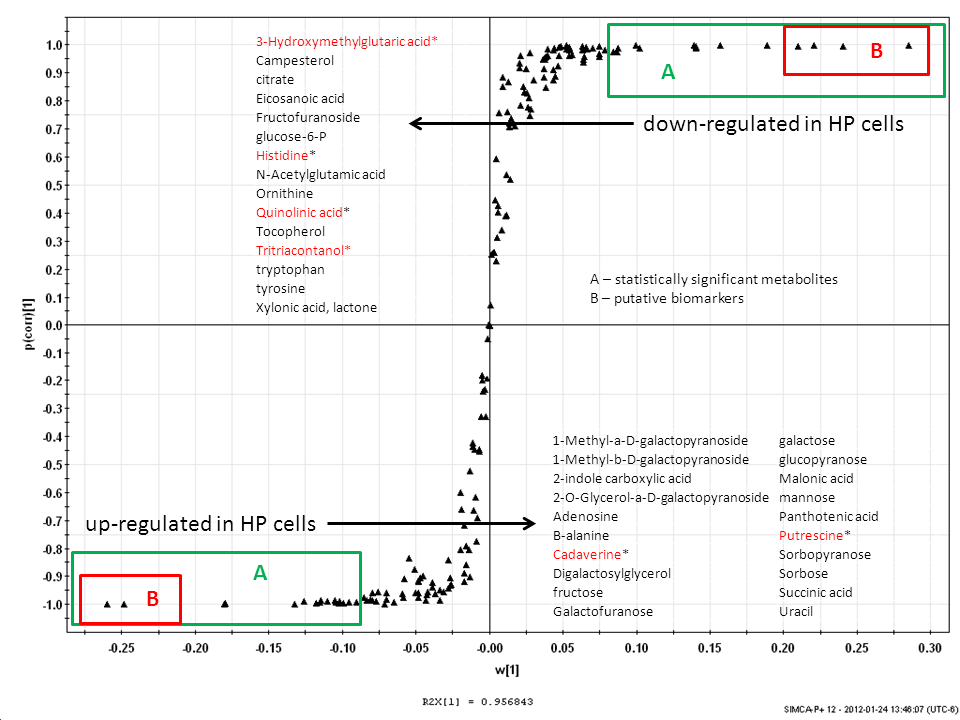


B


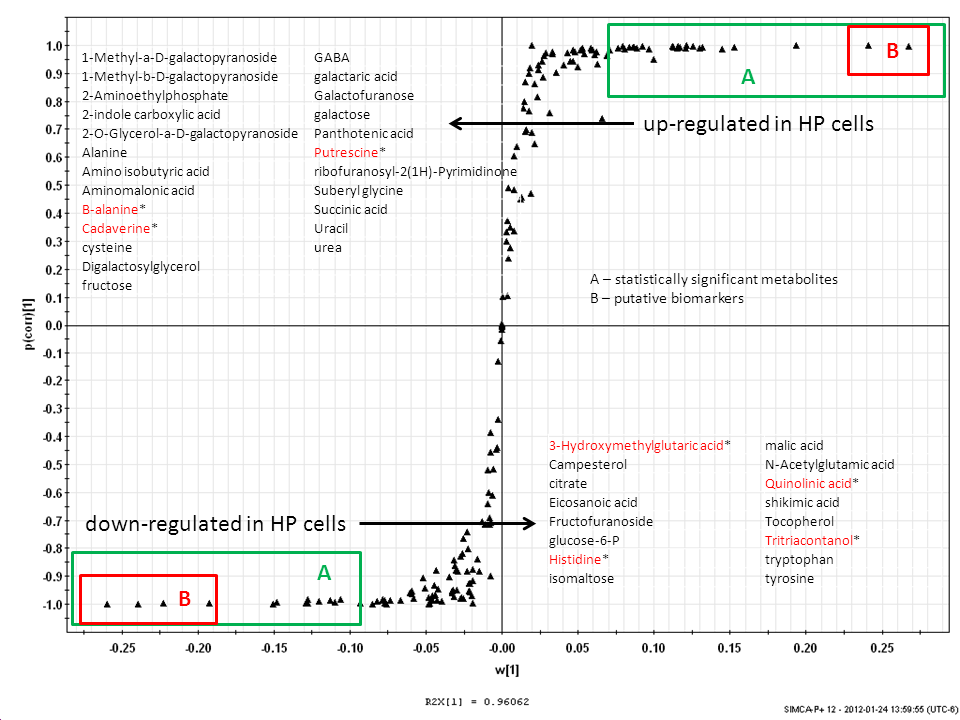


C


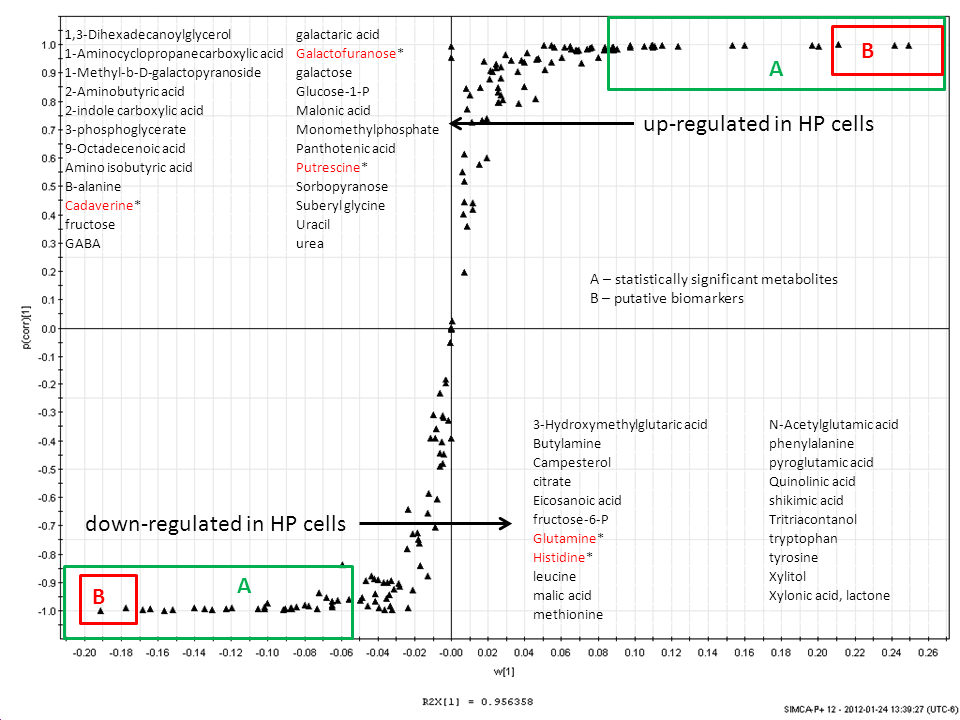


D

A

**Additional file 1: Figure S3**. The loading plots (S-plot) of the OPLS-DA results for the control and HP cell extracts on days 2 (A), 4 (B), and 6 (C). In the S-plot, each point represents a single metabolite (marker). The *x*-axis shows the variable contributions. The farther away a data point is from the 0 value, the more it contributes to sample variance. The *y*-axis shows the sample correlations within the same sample group. The farther away a metabolite is from the 0 value, the better is its correlation from injection to injection. As a result, the metabolites on both ends of the S-shaped curve represent the leading contributing ions from each sample group. The OPLS-DA is a multivariate analysis model which separates the systematic variation in X into two parts, one that is linearly related (and therefore predictive) to Y and one that is orthogonal to Y (unrelated); the Y-predictive/related part represents the between-class variation, the Y-orthogonal (ToPo) part constitutes the within-class variation. For details see: <https://mynotebook.labarchives.com/share/ulav72/MjIuMXwxNzEzMTkvMTcvVHJlZU5vZGUvMzg1Mzg2MTkxNHw1Ni4x>
