# Supplementary material for: Genetic manipulation of putrescine biosynthesis reprograms the cellular transcriptome and the metabolome
Source: BMC Plant Biol. 2016 May 18;16:113. doi: 10.1186/s12870-016-0796-2 (PMC4870780; doi:10.1186/s12870-016-0796-2)
Supplement: Additional file 2: — Methods for RNA extraction, cDNA preparation and labeling, microarray hybridization and processing, and metabolomic analysis. Supplemental data Microarrays: http://www.ncbi.nlm.nih.gov/geo/query/acc.cgi?acc=GSE79420. Metabolomic Data: https://mynotebook.labarchives.com/share/ulav72/MjIuMXwxNzEzMTkvMTcvVHJlZU5vZGUvMzg1Mzg2MTkxNHw1Ni4x. (PDF 451 kb) [file 12870_2016_796_MOESM2_ESM.pdf]

AUTHORS: PAGE A.F. ET AL. SUPPLEMENTAL MATERIAL

Experimental Procedures

RNA Extraction, cDNA preparation and labeling

The cells were collected by vacuum filtration on Miracloth, washed quickly with de-ionized water, and weighed. The cells were flash frozen in liquid nitrogen before storage at -80°C. Total RNA was extracted as described by Page and Minocha (2004). Following the removal of DNA (TURBO DNA-free^{TM} kit - Ambion Inc., Austin, TX) and quantification by NanoDrop (Thermo-Fisher), the RNA was reverse transcribed using a Superscript^{TM} Indirect cDNA Labeling System (Invitrogen, Carlsbad, CA). For this, 13.5 µg of RNA was incubated with 4 µL of oligo-dT primer in 36 µL reactions at 70°C for 5 min and then placed on ice. A master mix was prepared to minimize inter-sample variation and aliquoted into samples such that each received the following: First Strand Buffer (4 µL), 0.1 M DTT (1.5 µL), dNTPs (1.5 µL), RNase OUT (1.0 µL) and SuperScript III (2.0 µL). Reactions were carried out for 4 h at 46°C, and stopped by addition of 30 µL 1N NaOH and incubation at 70°C for 10 min. Reactions were neutralized by 30 µL of 1N HCl. Following reverse transcription, cDNA was purified using S.N.A.P. Columns (Invitrogen) according the manufacturer's instructions. Resultant cDNA was re-suspended in 10 µL Coupling Buffer.

Individual aliquots of Cy3 and Cy5 (RPN 5661, GE Healthcare, Piscataway, NJ) were re-suspended in 5 µL DMSO and then added to 5 µL of the cDNA in Coupling Buffer. Reactions were incubated at room temperature for 1 h in the dark before addition of 40 µL of sodium acetate (3 M, pH 5.2) and 500 µL of Loading Buffer. Following purification by S.N.A.P. column, the cDNA was eluted with 50 µL of DEPC-treated water and quantified by NanoDrop. Targets were prepared by combining 40 pmol each of labeled sample in an amber microfuge tube, which was flash-frozen in liquid nitrogen and dried in a vacuum centrifuge before storage at -20°C.

Microarray hybridization and processing

Targets were re-suspended in 55 µL of hybridization solution (50% formamide, 5X SSC, 0.1% SDS, and 0.1% BSA) and centrifuged at 14,000 ×g for 1 min before denaturation at 45°C for 5-10 min. Lifter slips (Thermo-Fisher) were rinsed in ddH_2O and then 100% ethanol. Corning

hybridization chambers (Corning Inc., Acton, MA) were warmed to $45^{o}C$ on a slide warmer, slides placed inside and lifter slips lowered into place on slides. Targets were added one drop at a time beside the end of lifter slips so as to allow capillary action to draw the solution between the lifter slip and the slide. Both humidifying wells in each chamber were filled with 15 µL of ddH_2O before assembly, and incubated for 36 h at $43^{o}C$ in a hybridization chamber.

Slides were removed from hybridization chamber and placed in 50 ml conical tubes containing 45 ml wash solution I (1X SSC, 0.2% SDS), and warmed to $45^{o}C$ to allow the lifter slip to fall off. They were then transferred to a new 50 ml tube, again containing 45 ml wash solution I and incubated for 15 min at $45^{o}C$ with gentle agitation. Slides were washed once at room temperature in wash solution II (0.1X SSC, 0.2% SDS) for 10 min, and twice in wash solution III (0.1X SSC) for 2 min each. Finally, the slides were immersed in ddH_2O for 10 s, 100% ethanol for 10 s, dried by centrifugation ($4^{o}C$, 2500 rpm, 2 min), and stored in the dark at room temperature.

Metabolomic analysis

For metabolite analysis, the cells (200 mg FW) were mixed with 3.0 ml of cold 40% methanol and stored at $-20^{o}C$ until analysis. The frozen cells were transported to the University of Illinois on dry ice. On arrival, the cells were vacuum dried and pulverized in 1.5 ml of 70% methanol by sonication for 30 min at $4^{o}C$ (2510 Branson Ultra-Sonicator - Danbury, CT) and incubated for 15 min at $60^{o}C$. Following centrifugation for 10 min at 10,000 x*g*, the supernatant was collected and the pellet was further extracted in 1.5 ml water at ambient temperature. The samples were centrifuged for 5 min at 10,000*g*, and water extracts were combined with previously collected methanol extracts. Extracts were dried under vacuum and processed as described under Materials and Methods.

LEGENDS TO SUPPLEMENTAL TABLES

Supplemental Table S1: Summary of changes in expression of genes involved in polyamine metabolism. For abbreviations see Figure 1. Sequences for AS, CARB, DAO, NAGK, NAGPR, NAOD, NAOGAcT, ODC, OTC and SPDS were not represented on the microarray.

Supplemental Table S2. Functional clustering of gene models showing significant ($p \leq 0.05$) differences (≥ 2 fold) between the HP and the control cell lines on both day 3 and day 5.

Supplemental Table S3. Functional clustering of gene models showing significant ($p \leq 0.05$) differences ($\geq$2 fold) between the HP and the control cell lines on day 3 only.

Supplemental Table S4. Functional clustering of gene models showing significant ($p \leq 0.05$) differences ($\geq$2 fold) between the HP and the control cell lines on day 5 only.

Supplemental Table S5: List of metabolites that were positively identified in poplar control and HP cell lines. ND = not detectable. Values that are significantly different ($P < 0.05$) in the HP cells from the corresponding control cells on a given day are marked in bold.

LEGENDS TO SUPPLEMENTAL FIGURES

Supplemental Fig. S1. The pathway for the biosynthesis of polyamines and related metabolites starting from the assimilation of nitrogen (Adapted from Majumdar et al. 2016).

Supplemental Figure S2. (A, B) - Quality control scatter plots showing expression level for data that passed CV and dye-swap tests. Red spots indicate data that passed statistical analysis for differential expression between the control and the HP cells.

Supplemental Figure S3. The loading plots (S-plot) of the OPLS-DA results for the control and HP cell extracts on days 2 (A), 4 (B), and 6 (C). In the S-plot, each point represents a single metabolite (marker). The *x*-axis shows the variable contributions. The farther away a data point is from the 0 value, the more it contributes to sample variance. The *y*-axis shows the sample correlations within the same sample group. The farther away a metabolite is from the 0 value, the better is its correlation from injection to injection. As a result, the metabolites on both ends of the S-shaped curve represent the leading contributing ions from each sample group. The OPLS-DA is a multivariate analysis model which separates the systematic variation in X into two parts, one that is linearly related (and therefore predictive) to Y and one that is orthogonal to Y (unrelated); the Y-predictive/related part represents the between-class variation, the Y-orthogonal (ToPo) part constitutes the within-class variation.

Supplemental Table S1: Summary of changes in expression of genes involved in polyamine metabolism. For abbreviations see Figure S1. Sequences for AS, CARB, DAO, NAGK, NAGPR, NAOD, NAOGAcT, ODC, OTC and SPDS were not represented on the microarray.

			3 d		5 d		3 and 5 d	
Gene name	Spots	Gene models	Spots passing CV + dye swap	Gene models passing CV + dye swap	Spots passing CV + dye swap	Gene models passing CV + dye swap	Spots passing CV + dye swap	Gene models passing CV + dye swap
ACCO	13	6	3	2	8	3	2	2
ACCS	10	7	6	4	9	6	6	4
AL	1	1	0	0	1	1	0	0
ARG	2	1	0	0	1	1	0	0
ADC	6	1	1	1	4	1	1	1
CARA	1	1	0	0	1	1	0	0
GAD	5	1	1	1	3	1	0	0
GOGAT	4	3	1	1	2	2	1	1
GS	28	7	12	4	21	6	11	4
LYSDC	5	5	0	0	1	1	0	0
NAGS	2	2	1	1	1	1	1	1
NAOAT	3	2	1	1	1	1	0	0
NAOD	0	0	0	0	0	0	0	0
NR	2	1	2	1	2	1	2	1
NiR	3	1	0	0	1	1	0	0
OAT	3	2	1	1	0	0	0	0
SAMDC	15	4	7	4	7	2	3	1
SPMS	3	2	1	1	0	0	0	0

Supplemental Table S2. Functional clustering of gene models showing significant (p<0.05) differences (>2 fold) between the HP and the control cell lines on both day 3 and day 5. **The specific Gene Ontology numbers associated with Molecular function/Biological process were obtained by searching with the gene names for Gene Ontology terms associated with plants using EMBL Quick go (http://www.ebi.ac.uk/QuickGO/).**
Bold = up regulated; normal = down regulated.

	**Specific GO number/s**	**Molecular function (MF) and/or Biological process (BP)**	**3 d Fold Change**	**5 d Fold Change**	**# ESTs for this model**
**Enzymatic**					
Glutathione-s-transferase	GO:0016740	MF: transferase activity	**2.87**	**2.21**	1
Acetyltransferase	GO:2000983	MF: regulation of acetyl-CoA:oxaloacetate acetyltransferase (isomerizing; ADP- phosphorylating) activity	**2.66**	**3.75**	1
Alcohol dehydrogenase 2	GO:0052933,GO:0052934	MF: alcohol dehydrogenase [cytochrome c(L)] activity, MF: alcohol dehydrogenase [cytochrome c] activity	-2.07	-2.31	1
Ubiquitin-conjugating enzyme E2-17 (UBC9)	GO:0035370	MF: catalyzes assembly of linked polyubiquitin chains	-2.39	-2.03	1
Cobalamine-independent methionine synthase	GO:0008705	MF:methionine synthase activity	-2.88	-2.69	1
Pectate lyase	GO:0030570	MF:pectic acid transeliminase activity and/or pectic acid lyase activity	-3.23	-2.53	4
Dolichyl-di-phosphooligosaccharide glycotransferase	GO:0004579	MF: dolichyl-diphosphooligosaccharide:protein-L-asparagine oligopolysaccharidotransferase activity	-5.94	-2.27	1
**Ribosomal/transcription/translation**					
Small nuclear ribonucleoprotein-like protein	GO:0006397	BP: Any process involved in the conversion of a primary mRNA transcript into one or more mature mRNA(s) prior to translation into polypeptide. mRNA maturation	**3.83**	**2.85**	1

F2D10.18 transcription regulator	GO:0001068	MF: Interacting selectively and non-covalently with a DNA region that regulates the transcription of a region of DNA	**3.68**	**2.04**	1
Histone H2A	GO:0043968,GO:0033522,GO:1990164, GO:0035518	BP: histoneH2A acetylation; histoneH2A ubiquitination; histoneH2A phosphorylation; histoneH2A monoubiquitination	**2.6**	**2.03**	2
Poly(A)-binding protein	GO:0003723, GO:0000398	MF: RNA binding, BP: nuclear mRNA splicing, via spliceosome	-2.16	-2.58	1
Translation initiation regulator like	GO:0031369	MF: polypeptide factor involved in the initiation of ribosome-mediated translation	-5.15	-4.29	1
**Membrane transport and osmoregulation**					
Plasma membrane intrinsic protein	GO:0005215, GO:0006810	MF: transporter activity, BP: transport	**9.95**	**7.12**	6
Aquaporin TIP3	GO:0015288, GO:0005215, GO:0006810	MF: porin activity, MF: transporter activity, BP: transport	-2.77	-2.24	1
Annexin Anx1	GO:0005509, GO:0005544	MF: calcium ion binding, MF: calcium-dependent phospholipid binding	-3.79	-2.36	1
**Stress/Wound associated**					
Wound induced protein	GO:0009611	BP: response to wounding	**7.84**	**4.09**	2
Basic chitinase	GO:0004568	MF:Catalysis of the hydrolysis of (1->4)-beta linkages of N-acetyl-D-glucosamine (GlcNAc) polymers of chitin and chitodextrins	**4.05**	**5.7**	2
Chitinase	GO:0004568	MF:Catalysis of the hydrolysis of (1->4)-beta linkages of N-acetyl-D-glucosamine (GlcNAc) polymers of chitin and chitodextrins	**3.11**	**7.14**	1
HEV1.2, hevein	GO:0008061	MF: chitin binding	**2.83**	**3.95**	1
Metallothionein 2a	GO:0046872	MF: Metal ion binding	**2.57**	**4.09**	2
Heat shock protein	GO:0031072	MF: Interacting selectively and non-covalently with a heat shock protein, any protein synthesized or activated in response to heat shock	**2.52**	**2.17**	1
Metallothionein 2b	GO:0046872	MF: Metal ion binding	**2.18**	**2.44**	1

Small heat shock protein - soybean	GO:0031072	MF: Interacting selectively and non-covalently with a heat shock protein, any protein synthesized or activated in response to heat shock	**1.01**	-1.05	2
Ascorbate peroxidase	GO:0004601, GO:0006979	MF: peroxidase activity, BP: response to oxidative stress	-2.21	-2.18	1
Pathogenesis-related protein	GO:0006952	MF: Biological process: defense response	-2.45	-3.77	1
Stress related protein	GO:0006950	BP: response to any kind of stress	-2.51	-2.31	1
Cationic peroxidase	GO:0005506, GO:0016491,GO:0004601, GO:0006979	MF: iron ion binding, MF: oxidoreductase activity, MF: peroxidase activity, BP: response to oxidative stress	-5.39	-4.07	17
**Cell wall**					
Cell wall-plasma membrane linker protein	GO:0005199	MF: structural constituent of cell wall	-2.04	-2.29	1
3,5-epimerase/4-reductase	GO:0003824,GO:0008831,GO:0051287	MF: catalytic activity, MF: dTDP-4-dehydrorhamnose reductase activity, MF: NAD binding	-3.29	-2.25	1
Fasciclin-like AGP 10	GO:0007155	BP: cell adhesion	-4.84	-4.7	1
Extensin like protein - black poplar	GO:0010409	BP: extensin metabolic process	-5.87	-5.81	5
Extensin like protein - black poplar	GO:0010409	BP: extensin metabolic process	-8	-8.46	8
Extensin like protein	GO:0010409	BP: extensin metabolic process	-16.28	-18.46	12

Supplemental Table S3. Functional clustering of gene models showing significant (p<0.05) differences (>2 fold) between the HP and the control cell lines on day 3 only. **Bold** = up regulated; normal = down regulated.

	**Specific GO number/s**	**Molecular function and/or biological process**	**Fold Change**	**# ESTs for this model**
**Enzymatic**				
NADH dehydrogenase subunit 1	GO:0003954	MF: NADH2 dehydrogenase activity	**3.29**	1
Glyceraldehyde-3-phosphate dehydrogenase	GO:0008943	MF: Glyceraldehyde-3-phosphate dehydrogenase activity	**3.25**	1
Ubiquinol--cytochrome c reductase	GO:0008121	MF: Catalysis of the transfer of a solute or solutes from one side of a membrane to the other according to the reaction	**2.61**	1
Beta-amylase	GO:0016161, GO:0000272	MF: beta-amylase activity; BP: polysaccharide catabolism	**2.37**	1
Fructose-bisphosphate aldolase	GO:0004332, GO:0006096	MF: fructose-bisphosphate aldolase activity; BP: glycolysis	-2.07	3
Phenylcoumaran benzylic ether reductase	GO:0032442	MF: Catalysis of the NADPH-dependent 7-O-4' reduction of phenylcoumaran lignans to the corresponding diphenols	-2.15	1
Serine peptidase	GO:0008236	MF: Catalysis of the hydrolysis of peptide bonds in a polypeptide chain by a catalytic mechanism that involves a catalytic triad consisting of a serine nucleophile that is activated by a proton relay involving an acidic residue (e.g. aspartate or glutamate) and a basic residue (usually histidine).	-2.17	1
Peptidyl-prolyl cis-trans isomerase	GO:0003755	MF: Peptidyl-prolyl cis-trans isomerase activity	-2.18	2
RUB1 conjugating enzyme	GO:0008642, GO:0006512	MF: ubiquitin-like activating enzyme activity, BP: ubiquitin cycle	-2.24	2
Methionine synthase	GO:0008168, GO:0009086	MF: methyltransferase activity, BP: methionine biosynthesis	-2.25	1

Photosystem II 32 kDa protein	GO:0019684	BP:photosynthesis, light reaction	-2.28	1
Glucose-6-phosphate dehydrogenase	GO:0004345	MF: glucose-6-phosphate 1-dehydrogenase activity	-2.37	1
Pectate lyase	GO:0030570	MF:pectic acid transeliminase activity and/or pectic acid lyase activity	-2.39	1
Polygalacturonase-like protein	GO:0004650	MF:random hydrolysis of (1->4)-alpha-D-galactosiduronic linkages in pectate and other galacturonans	-2.43	1
Ubiquitin-conjugating enzyme 9	GO:0035370	MF: catalyzes assembly of linked polyubiquitin chains	-2.63	3
Aspartic proteinase	GO:0070001	MF: aspartic-type peptidase activity	-2.74	2
Cytosolic phosphoglucomutase	GO:0004614	MF:Catalysis of the conversion reaction: alpha-D-glucose 1-phosphate = alpha-D-glucose 6-phosphate	-3.19	1
Formate dehydrogenase	GO:0008863	MF: formate dehydrogenase (NAD+) activity	-3.31	1
P protein, component of aminomethyltransferase	GO:0004047	MF:aminomethyltransferase activity	-3.63	1
Malate dehydrogenase	GO:0016615	MF: Catalysis of the reversible conversion of pyruvate or oxaloacetate to malate	-3.85	1
Wax synthase isoform 1	GO:0047196, GO:0016740	MF: long-chain-alcohol O-fatty-acyltransferase activity, MF: transferase activity	-3.86	1
Pectate lyase	GO:0030570	MF: pectin transeliminase activity or pectic acid lyase activity	-4.45	1
**Ribosomal/transcription/translation**				
Acidic ribosomal protein P1a	GO:0003735, GO:0006610	MF:contributes to the structural integrity of the ribosome, BP: ribosomal protein import into nucleus	**3.15**	1
Ribosomal protein L9	GO:0003735, GO:0006610	MF:contributes to the structural integrity of the ribosome, BP: ribosomal protein import into nucleus	**3.1**	1
Histone H2A	GO:0043968, GO:0033522, GO:1990164, GO:0035518	BP: histoneH2A acetylation; histoneH2A ubiquitination; histoneH2A phosphorylation; histoneH2A monoubiquitination	**2.92**	2

Histone H1	GO:0018024	MF: histone H1-specific S-adenosylmethionine:protein-lysine N-methyltransferase activity	**2.38**	1
Acidic ribosomal protein P1a	GO:0003735, GO:0006610	MF:contributes to the structural integrity of the ribosome, BP: ribosomal protein import into nucleus	**2.15**	1
Ribosomal protein	GO:0003735, GO:0006610	MF:contributes to the structural integrity of the ribosome, BP: ribosomal protein import into nucleus	**2.14**	1
Ribosomal protein S19	GO:0003735, GO:0006610	MF:contributes to the structural integrity of the ribosome, BP: ribosomal protein import into nucleus	**2.02**	1
Ribosomal protein S4	GO:0003735, GO:0006610	MF:contributes to the structural integrity of the ribosome, BP: ribosomal protein import into nucleus	-2.07	1
Ribosomal protein L7A	GO:0003735, GO:0006610	MF:contributes to the structural integrity of the ribosome, BP: ribosomal protein import into nucleus	-2.17	1
Histone protein 60	-	-	-2.28	1
Ribosomal protein L10	GO:0003735, GO:0006610	MF:contributes to the structural integrity of the ribosome, BP: ribosomal protein import into nucleus	-4.24	1
Transcription factor-like	GO:0001070	MF:Interacting selectively and non-covalently with an RNA sequence to modulate transcription	-9.36	1
**Membrane transport and osmoregulation**				
Plasma membrane intrinsic protein	GO:0005215, GO:0006810	MF: transporter activity, BP: transport	**10.08**	1
Hydrogen-transporting ATP synthase activity	GO:0046933	MF: hydrogen ion transporting two-sector ATPase activity	**2.39**	1
Plasma membrane intrinsic protein	GO:0005215, GO:0006810	MF: transporter activity, BP: transport	**2.27**	1
Plasma membrane intrinsic protein	GO:0005215, GO:0006810	MF: transporter activity, BP: transport	-2.06	1
Annexin	GO:0005509, GO:0005544	MF: calcium ion binding, MF: calcium-dependent phospholipid binding	-2.88	2
Aquaporin	GO:0015288, GO:0005215	MF: porin activity, MF: transporter activity	-3.08	1
Osmotin	GO:0006970	BP: osmotic stress response	-5.46	2

**Stress/Wound associated**				
Heat shock protein	GO:0031072	MF: protein synthesized or activated in response to heat shock	**3.85**	1
Cytosolic class II low MW heat shock protein	GO:0031073	MF: protein synthesized or activated in response to heat shock	**2.86**	1
Heat shock protein	GO:0031072	MF: protein synthesized or activated in response to heat shock	**2.29**	1
Proteinase inhibitor se60-like protein (defence)	GO:1900277	BP: Any process that stops, prevents or reduces the frequency, rate or extent of proteinase activated receptor activity	-2.06	1
Dehydration stress-induced protein	GO:0009414	BP: response to water deprivation	-2.06	1
Ascorbate peroxidase	GO:0004601, GO:0006979	MF: peroxidase activity, BP: response to oxidative stress	-2.13	1
Cationic peroxidase 2	GO:0005506, GO:0016491, GO:0004601, GO:0006979	MF: iron ion binding, MF: oxidoreductase activity, MF: peroxidase activity, BP: response to oxidative stress	-2.2	1
Pathogenesis-related protein	GO:0006952	BP: defense response	-3.97	1
**Cell wall**				
3,5-epimerase/4-reductase	GO:0003824, GO:0008831, GO:0051287	MF: catalytic activity, MF: dTDP-4-dehydrorhamnose reductase activity, MF: NAD binding	-2.83	1

Supplemental Table S4. Functional clustering of gene models showing significant (p<u><</u>0.05) differences (<u>></u>2 fold) between the HP and the control cell lines on day 5 only. **Bold** = up regulated; normal = down regulated.

	**Specific GO number/s**	**Molecular function and/or biological process**	**Fold Change**	**# ESTs this model**
**Enzymatic**				
NADH dehydrogenase ubiquinone	GO:0008137	MF: Catalysis of the reaction: NADH + H+ + ubiquinone = NAD+ + ubiquinol	**3.87**	2
Tropinone reductase	GO:0050358	MF: tropinone (psi-tropine-forming) reductase activity	**2.96**	3
Gamma-glutamylcysteine synthetase	GO:0004357	gamma-glutamyl-L-cysteine synthetase activity	**2.71**	1
Alternative oxidase	GO:0009916, GO:0010230	MF: alternative oxidase activity, BP: alternative respiration	**2.68**	1
Cinnamate 4-hydroxylase	GO:0016710	MF: t-cinnamic acid hydroxylase activity, Catalysis of the reaction: trans-cinnamate + NADPH + H+ + O2 = 4-hydroxycinnamate + NADP+ + H2O	**2.35**	1
Glycerophosphoryl diester phosphodiesterase family protein	GO:0008889	MF:glycerophosphoryl diester phosphodiesterase activity<br>Catalysis of the reaction: a glycerophosphodiester + H2O = an alcohol + sn-glycerol 3-phosphate	**2.12**	1
E2, ubiquitin-conjugating enzyme	GO:0008642, GO:0006512, GO:0032355	MF: ubiquitin-like activating enzyme activity, BP: ubiquitin cycle, BP: Cellular response to estradiol stimulus	**2.08**	1
Laccase	GO:0005507, GO:0016491, GO:0065009	MF: copper ion binding, MF: oxidoreductase activity, BP: regulation of molecular function	-2.01	1
UDP-glucoronosyl/UDP-glucosyl transferase family protein	GO:0035251, GO:0097359	MF:Catalysis of the transfer of a glucosyl group from UDP-glucose to an acceptor molecule, BP:UDP-glucosylation	-2.05	1

Acyl-CoA independent ceramide synthase	GO:0097001, GO:0006672	MF:ceramide binding, BP:ceramide metabolic process and pathways involving ceramides	-2.18	1
Chalcone isomerase	GO:0045430, GO:0009714	MF:Catalysis of the isomerase reaction: a chalcone = a flavanone, chalcone metabolic process	-2.32	1
Adenosylhomocysteinase	GO:0004013, GO:0006730	MF: adenosylhomocysteinase activity, BP: one-carbon compound metabolism	-2.44	1
NADPH-cytochrome P450 oxydoreductase isoform 3	GO:0015034	MF: Cytochrome P450 activity	-2.9	1
**Ribosomal/transcription/translation**				
Histone H2A	GO:0043968, GO:0033522, GO:1990164, GO:0035518	BP: histoneH2A acetylation; histoneH2A ubiquitination; histoneH2A phosphorylation; histoneH2A monoubiquitination	2.44	1
DNA-binding protein	GO:0003677, GO:0008642, GO:0006512	MF: DNA binding, MF: ubiquitin-like activating enzyme activity, BP: ubiquitin cycle	2.06	1
Ribosomal protein L34	GO:0003735, GO:0006610	MF:contributes to the structural integrity of the ribosome, BP: ribosomal protein import into nucleus	-2.04	1
Ribosomal protein L30	GO:0003735, GO:0006610	MF:contributes to the structural integrity of the ribosome, BP: ribosomal protein import into nucleus	-2.08	1
Ribosomal protein S25	GO:0003735, GO:0006610	MF:contributes to the structural integrity of the ribosome, BP: ribosomal protein import into nucleus	-2.12	2
Ribosomal protein S4	GO:0003735, GO:0006610	MF:contributes to the structural integrity of the ribosome, BP: ribosomal protein import into nucleus	-2.15	1
Nucleolar protein	GO:0034503	BP: Process in which a protein is transported to, or maintained at, the rDNA repeats on a chromosome in the nucleolus	-2.21	1
Ribosomal protein L17-1	GO:0003735, GO:0006610	MF:contributes to the structural integrity of the ribosome, BP: ribosomal protein import into nucleus	-2.22	1
Ribosomal protein S11	GO:0003735, GO:0006610	MF:contributes to the structural integrity of the ribosome, BP: ribosomal protein import into nucleus	-2.22	1
Ribosomal protein S11	GO:0003735, GO:0006610	MF:contributes to the structural integrity of the ribosome, BP: ribosomal protein import into nucleus	-2.24	2

Eukaryotic translation initiation factor 2 alpha subunit eIF2	GO:0003743	MF: functions in the initiation of ribosome-mediated translation of mRNA into a polypeptide	-2.31	1
Ribosomal protein S30	GO:0003735, GO:0006412	MF: structural constituent of ribosome, BP: protein biosynthesis	-2.43	2
Transcription factor LIM	GO:0001070	MF: Interacting selectively and non-covalently with RNA in order to modulate transcription.	-2.46	1
**Membrane transport and osmoregulation**				
Imbibition protein	-	-	**2.39**	1
Aquaporin	GO:0015288, GO:0005215	MF: porin activity, MF: transporter activity	**2.25**	1
Plasma membrane intrinsic protein	GO:0005215, GO:0006810	MF: transporter activity, BP: transport	**2.23**	1
Plasma membrane intrinsic protein	GO:0005215, GO:0006810	MF: transporter activity, BP: transport	**2.05**	1
Vacuolar V-H+ATPase subunit E	GO:0070072	BP: proton-transporting two-sector ATPase complex that couples ATP hydrolysis to the transport of protons across the vacuolar membrane	**2.04**	1
Vacuolar V-H+ATPase subunit E	GO:0070072	BP: proton-transporting two-sector ATPase complex that couples ATP hydrolysis to the transport of protons across the vacuolar membrane	**2.04**	1
Vacuole-associated annexin VCaB42	GO:0005509	MF: calcium ion binding ,calcium-dependent phospholipid binding	-2.05	1
**Stress/Wound associated**				
Peroxidase ATPA2	GO:0005506, GO:0016491, GO:0004601, GO:0006979	MF: iron ion binding, MF: oxidoreductase activity, MF: peroxidase activity, BP: response to oxidative stress	**2.5**	1
Metallothionein 2b	GO:0046872	MF: Metal ion binding	**2.35**	3
Beta-1,3-glucanase	GO:0033903	MF: endo-1,3(4)-beta-glucanase activity	**2.3**	1
Metallothionein 1b	GO:0046872	MF: Metal ion binding	**2.13**	26
Metallothionein 1a	GO:0046872	MF: Metal ion binding	**2.11**	2

Metallothionein 1a	GO:0046872	MF: Metal ion binding	**2.1**	1
Metallothionein 2a	GO:0046872	MF: Metal ion binding	**2.06**	1
Cold stress protein SRC1	GO:0009409	BP:response to cold stress	**2.05**	1
Metallothionein 1a	GO:0046872	MF: Metal ion binding	**2.02**	1
SCOF-1	-	-	-3.13	1
**Cell Wall**				
3,5-epimerase/4-reductase	GO:0003824, GO:0008831, GO:0051287	MF: catalytic activity, MF: dTDP-4-dehydrorhamnose reductase activity, MF: NAD binding	-2.36	1
Xyloglucan endotransglycosylase	GO:0016762	MF: xyloglucan endotransglycosylase activity	-2.71	2

Supplemental Table S5: List of metabolites that were positively identified in poplar control and HP cell lines. ND = not detectable. GC/MS detected a total of 645 compounds in the control cell line and 680 in the HP cell line. Of these, 190 and 178 compounds were positively identified in the control and the HP cells, respectively with the total number of positively identified compounds being >200. Values that are significantly different (P < 0.05) in the HP cells from the corresponding control cells on a given day are marked in **bold**.

Relative conc. g FW^{-1}	Con-2d	HP-2d	Con-4d	HP-4d	Con-6d	HP-6d
Alcohols						
Erythritol	7.6±1.2	**4.1±0.7**	6.8±0.3	7.1±0.8	6.5±0.6	7.1±0.5
Galactitol	2.9±0.2	**ND**	1.4±0.2	**ND**	ND	ND
Glycerol	182.2±12.8	**135.0±3.9**	154.1±20.9	134.6±18.0	119.6±10.2	188.2±19
Glycerol-2-P	0.7±0.1	**ND**	1.7±0.2	**ND**	0.4±0.1	**ND**
Glycerol-3-p	17.3±4.1	14.7±3.1	11.1±1.5	13.7±4.3	4.3±0.4	8.3±1.5
Inositol	2247.2±199.6	2601.0±280.1	3288.8±251.3	3133.5±95.1	3237.0±262.0	3718.6±97.5
Inositol-P	30.5±2.9	**16.0±3.5**	33.7±3.3	**12.8**±1.2	26.6±3.5	25.4±3.5
Maltitol	1.0±0.2	**ND**	1.1±0.2	**ND**	ND	ND
Mannitol	3.3±0.2	3.4±0.4	3.8±0.3	3.4±0.3	5.0±0.7	4.9±0.3
Octadecanol	1.0±0.1	**0.7±0.1**	1.2±0.2	1.1±0.2	1.2±0.3	1.0±0.1
Ribitol	3.1±0.8	**9.5±0.6**	8.5±1.2	6.0±0.4	9.3±0.8	6.7±1.5
Sorbitol	7.0±1.2	**4.1±0.7**	6.2±2.2	6.4±1.3	6.4±2.9	2.6±0.3
Sorbitol-6-P	5.2±0.8	**3.1±0.1**	7.4±1.5	3.8±0.3	7.1±0.8	**3.9**±0.3
Tetracosanol	0.3±0.1	0.2±0.0	ND	**0.2**±0.1	ND	ND
Tetratriacontanol	1.2±0.2	1.2±0.2	1.3±0.2	1.5±0.3	1.3±0.3	1.2±0.4
Threitol	1.4±0.4	**3.3±0.5**	3.8±0.6	2.7±0.6	3.6±0.5	3.0±0.5
Triacontanol	0.4±0.1	0.5±0.1	0.2±0.2	0.5±0.1	0.5±0.1	0.5±0.0
Tritriacontanol	8.7±1.9	**0.4±0.1**	10.1±2.6	**0.3**±0.0	6.1±0.6	**0.5**±0.1
Xylitol	3.7±0.5	3.4±0.3	9.3±1.4	**3.8**±0.7	10.5±1.3	**2.3**±0.3
Lipids						
1,3-Dihexadecanoylglycerol	0.7±0.2	**1.4±0.2**	0.9±0.1	**2.7**±0.4	0.8±0.1	**3.0**±0.3
11,14,17-Eicosatrienoic acid	2.8±0.1	2.9±0.2	3.9±0.2	3.0±0.4	3.4±0.3	2.3±0.3

1-Monohexadecanoylglycerol	8.1±0.5	**2.8±0.2**	4.0±0.7	5.4±1.2	3.0±0.4	**5.9**±0.9
9,12,15-Octadecatrienoic acid	56.1±2.2	49.5±2.2	74.4±6.3	101.6±13.9	76.4±2.4	85.8±4.7
9,12-Octadecadienoic acid	75.6±3.5	**28.3±3.6**	63.2±2.1	**34.3**±2.7	66.8±4.4	52.6±8.3
9-Octadecenoic acid	12.0±2.3	**3.9±0.1**	5.5±1.3	**13.7**±2.5	1.5±0.5	**24.6**±1.4
Docosanoic acid	2.9±0.2	**1.4±0.1**	3.1±0.2	**1.9**±0.3	2.9±0.1	**1.8**±0.1
Dodecanoic acid	1.2±0.1	1.4±0.2	1.4±0.4	1.7±0.3	1.7±0.5	1.5±0.3
Eicosanoic acid	1.3±0.3	**0.1±0.0**	1.2±0.4	**0.1**±0.0	1.3±0.4	**0.1**±0.0
Heptadecanoic acid	1.9±0.4	1.8±0.3	1.6±0.3	1.7±0.3	1.9±0.2	1.5±0.3
Heptanoic acid	0.6±0.1	0.7±0.1	0.6±0.1	0.7±0.1	0.5±0.1	0.7±0.0
Hexacosanoic acid	0.5±0.1	0.6±0.1	0.5±0.1	0.5±0.0	0.4±0.1	0.5±0.1
Hexadecanoic acid	94.8±10.8	**56.1±5.9**	97.1±9.2	**71.3**±3.5	95.5±7.6	87.9±9.2
Hexadecanoic acid, 1-[[oxy]methyl]-1,2-ethanediyl	2.9±0.2	**ND**	3.4±0.1	**ND**	2.7±0.3	**ND**
Nonanoic acid	7.6±2.0	**5.0±0.8**	7.8±1.6	5.3±1.0	4.3±0.6	**1.6**±0.5
Octacosanoic acid	0.5±0.1	**0.3±0.1**	0.8±0.1	0.5±0.1	0.7±0.0	0.4±0.1
Octadecanoic acid	33.0±2.3	27.8±3.0	32.9±2.0	33.3±2.6	30.5±2.5	28.5±3.1
Oleanitrile	1.6±0.3	1.7±0.4	1.4±0.4	**ND**	ND	ND
Pentacosanoic acid	0.5±0.1	0.3±0.0	0.7±0.1	**0.3**±0.1	0.3±0.0	0.3±0.0
Pentadecanoic acid	1.6±0.3	**ND**	1.7±0.0	**ND**	1.8±0.2	**ND**
Tetracosanoic acid	4.0±0.7	**ND**	3.7±0.4	**ND**	2.8±0.6	ND
Triacontanoic acid	0.9±0.1	**0.5±0.1**	1.1±0.1	**0.5**±0.0	1.0±0.2	0.5±0.1
Tricosanoic acid	0.4±0.1	0.5±0.0	0.4±0.2	0.5±0.1	0.4±0.2	0.4±0.1
Nitrogenous metabolites						
2-Methylserine	2.5±0.2	2.8±0.2	1.2±0.1	**2.7**±0.2	1.6±0.2	**4.7**±0.6
2-Aminobutyric acid	1.3±0.1	1.5±0.1	1.7±0.3	**3.9**±0.1	1.7±0.2	**5.5**±0.2
2-Aminoethylphosphate	3.7±0.9	7.6±2.0	0.8±0.1	**5.3**±0.3	ND	**3.7**±0.8
5-Methylthioadenosine	10.6±2.4	5.7±1.0	8.3±0.6	**4.1**±0.5	6.9±0.2	**4.9**±0.2
Adenine	3.6±0.5	2.8±0.4	2.8±0.3	2.7±0.7	3.1±0.4	2.6±0.3
Adenosine	14.0±2.4	**46.2±8.1**	16.1±1.5	**48.5**±4.3	17.9±1.0	**44.9**±6.5
Adenosine-5-P	32.6±3.8	23.5±3.1	28.0±1.6	25.6±2.2	16.2±1.9	**22.7**±1.2
Alanine	235.2±11.5	**693.1±97.3**	207.2±27.0	**747.8**±80.6	345.3±33.0	**1004.4**±89.3

Amino isobutyric acid	5.3±0.9	10.0±2.2	2.6±0.3	**10.7**±1.6	3.2±0.4	**15.2**±0.8
Aminomalonic acid	1.0±0.2	1.3±0.4	0.4±0.1	**1.4**±0.2	0.7±0.1	1.2±0.2
Asparagine	40.3±2.0	39.4±2.6	37.6±1.2	27.8±2.0	35.2±4.3	21.9±1.9
Aspartic acid	157.5±18.6	**91.7±6.3**	99.1±3.0	89.0±3.0	93.0±7.9	127.0±11.7
β-Alanine	14.5±2.5	**67.1±6.2**	3.4±0.2	**57.0**±5.4	3.9±0.5	**36.5**±1.3
Butylamine	0.7±0.1	**0.2±0.0**	0.6±0.1	**0.2**±0.0	1.0±0.2	**0.2**±0.1
Cadaverine	0.5±0.1	**19.9±2.2**	0.5±0.0	**19.5**±2.2	0.5±0.1	**18.0**±1.7
Cysteine	4.0±1.0	5.2±0.6	2.2±0.3	**7.5**±0.3	9.0±1.0	7.0±0.6
Cytosine	0.2±0.1	0.2±0.0	0.3±0.1	0.2±0.0	0.2±0.0	0.2±0.1
Ethanolamine	184.8±10.0	**334.7±51.3**	165.6±35.6	**272.9**±16.1	159.4±6.9	**280.3**±46.3
GABA (γ-aminobutyric acid)	154.2±24.4	**405.1±13.4**	120.1±10.9	**455.8**±8.8	53.2±6.6	**446.0**±33.9
Glutamic acid	430.6±68.4	**152.6±32.5**	235.0±11.5	**129.8**±39.0	242.4±31.6	**136.0**±17.5
Glutamine	38.5±7.8	**15.7±3.4**	26.9±3.7	**12.9**±3.2	163.5±37.6	13.8±3.2
Glycine	176.4±33.9	**344.7±58.8**	188.7±3.7	203.2±42.6	208.1±34.0	296.6±37.7
Guanine	0.2±0.0	**ND**	0.2±0.0	**ND**	0.2±0.0	ND
Histidine	38.0±1.4	**1.7±0.3**	28.3±3.1	**1.7**±0.2	30.4±3.0	**2.0**±0.1
Homocysteine	0.4±0.1	0.3±0.0	0.5±0.0	**0.1**±0.0	0.4±0.1	**0.1**±0.0
Homoserine	5.2±0.2	4.3±0.4	3.5±0.3	**2.3**±0.2	2.9±0.1	3.3±0.5
Hydroxylamine	5.5±0.2	**13.8±1.4**	3.5±0.4	**9.9**±2.3	4.1±0.8	**11.2**±3.3
Inosine	2.4±0.3	**ND**	4.7±0.2	**ND**	1.4±0.3	ND
Isoleucine	34.5±2.2	25.9±1.8	30.4±1.9	28.0±2.8	83.7±10.2	78.7±8.4
Leucine	83.5±2.4	**34.5±7.9**	64.6±5.7	**47.1**±0.4	257.8±30.6	**76.5**±6.5
Lysine	39.8±5.4	**16.4±3.3**	34.1±3.7	27.1±2.0	60.4±5.4	**21.8**±1.3
Methionine	13.4±3.2	**4.6±0.3**	11.9±0.2	**3.9**±0.6	11.6±1.8	**3.5**±0.2
N-Acetylglutamic acid	15.7±3.4	**4.7±0.4**	24.0±1.3	**6.2**±0.6	21.6±2.5	**6.1**±0.4
N-Acetyl-serine	3.6±0.5	4.4±0.2	5.4±0.8	3.7±0.7	8.0±0.9	**4.1**±0.6
Ornithine	1.4±0.2	**0.3±0.1**	0.8±0.1	0.5±0.1	0.5±0.0	0.5±0.1
Phenylalanine	36.3±3.3	**17.1±2.8**	32.6±0.8	**17.5**±2.7	109.9±13.2	**20.8**±3.1
Pipecolic acid	1.2±0.0	2.5±0.5	1.6±0.2	**2.6**±0.1	1.5±0.2	**2.6**±0.3
Proline	48.1±6.4	**28.6±7.9**	25.8±3.2	23.3±3.0	30.3±2.8	33.0±2.1
Putrescine	18.4±2.1	**691.8±13.1**	15.8±4.2	**742.1**±10.3	41.0±3.1	**771.0**±39.0

Pyroglutamic acid	82.2±4.5	**44.4±2.7**	78.3±1.8	**59.4**±0.4	283.8±45.1	**68.8**±7.6
Serine	248.3±23.3	**357.0±28.6**	116.0±15.4	**272.5**±10.2	179.6±32.7	155.3±39.1
Spermidine	19.1±1.7	17.9±2.9	13.9±4.9	21.3±2.1	14.4±2.7	20.8±2.2
Suberyl glycine	1.8±0.2	**2.9±0.2**	0.5±0.0	**2.9**±0.3	0.6±0.1	**2.4**±0.4
Threonine	231.0±32.4	**95.3±4.7**	189.1±10.3	**107.2**±4.6	140.2±49.5	97.3±7.8
Thymine	0.3±0.1	**0.1±0.0**	0.3±0.0	**0.1**±0.0	0.1±0.0	0.1±0.0
Tryptophan	57.0±3.4	**6.3±0.7**	37.0±9.5	**7.8**±0.3	47.1±3.2	**5.3**±0.3
Tyrosine	37.9±3.1	**2.6±0.3**	35.1±2.6	**3.9**±0.3	43.2±4.5	**5.2**±0.8
Uracil	2.8±0.1	**18.1±2.0**	2.3±0.3	**15.9**±2.4	2.4±0.1	**9.7**±0.5
Urea	1.6±0.2	**5.0±0.6**	1.6±0.1	**9.2**±0.9	1.6±0.1	**7.8**±0.6
Uric acid	3.3±0.4	2.2±0.4	1.6±0.1	2.1±0.2	1.6±0.1	1.4±0.2
Uridine	11.8±1.7	**23.1±2.5**	17.6±2.7	22.6±2.3	17.3±1.3	22.2±0.5
Valine	63.0±5.0	**36.0±0.8**	59.8±3.1	**41.5**±1.5	70.3±5.0	**45.0**±7.8
Organic acids						
1-Aminocyclopropanecarboxylic acid	30.6±2.3	**44.6**±4.5	46.2±4.3	**85.8**±7.1	29.2±3.5	**102.9**±14.7
2,4,5-Trihydroxypentanoic acid	1.8±0.2	1.8±0.2	1.4±0.4	1.6±0.3	1.0±0.1	1.3±0.3
2,4-Hydroxybutanoic acid	2.5±0.2	2.3±0.4	2.4±0.3	1.7±0.3	1.0±0.3	1.7±0.5
2-Indole carboxylic acid	7.7±0.7	**29.7±3.1**	6.9±1.2	**35.5**±3.0	7.6±0.9	**33.0**±2.3
2-Keto-gluconic acid	110.9±5.4	**56.1±2.7**	174.0±17.1	**56.0**±6.9	160.8±32.1	**62.0**±10.2
2-Methylbenzoic acid	0.6±0.1	0.6±0.2	0.8±0.2	0.5±0.0	1.0±0.1	0.7±0.2
3,4-Dihydroxybutanoic acid	0.4±0.1	0.3±0.1	0.4±0.1	0.4±0.1	0.5±0.1	0.4±0.0
3-Hydroxymethylglutaric acid	290.3±63.2	**10.0±1.5**	270.0±19.2	**8.4**±0.5	66.4±3.8	**12.1**±1.8
4-Hydroxybutanoic acid	1.9±0.3	2.9±0.5	1.9±0.1	2.0±0.3	2.1±0.1	2.0±0.3
Aconitic acid	3.7±0.2	**ND**	7.3±1.1	**ND**	6.2±1.0	**ND**
α-Ketoglutaric acid	4.0±0.1	**1.8±0.4**	3.4±0.3	2.6±0.2	6.0±1.0	3.8±0.1
Arabinonic acid, lactone	1.1±0.3	**3.3±0.2**	1.7±0.3	**3.2**±0.2	1.2±0.2	**3.0**±0.1
Ascorbic acid	73.3±4.7	66.7±1.8	66.5±3.3	**45.0**±2.2	17.9±1.2	14.9±0.8
Benzoic acid	0.7±0.1	**ND**	0.7±0.1	**ND**	0.6±0.2	ND
Citric acid	141.1±9.5	**20.0±2.9**	125.4±10.5	**19.5**±1.8	77.4±5.4	**18.8**±2.0
Dehydroascorbic acid	13.8±2.7	12.2±2.4	22.6±2.6	27.9±2.0	1.9±0.5	2.7±0.2

Fumaric acid	13.7±0.9	19.8±2.6	28.4±4.1	32.6±1.3	39.3±8.9	28.9±3.0
Galactaric acid	1.3±0.3	**3.6±0.5**	1.9±0.2	**6.9**±0.5	1.8±0.3	**5.7**±1.4
Galactonic acid	6.8±0.6	**15.7±3.1**	7.1±0.4	**15.1**±3.8	6.3±1.0	**16.3**±1.3
Glucaric acid	1.6±0.7	**3.3±0.6**	2.2±0.3	3.2±0.3	1.5±0.4	**3.5**±0.2
Gluconic acid	2.7±0.1	**4.5±0.6**	2.3±0.2	**4.6**±0.4	12.2±2.1	**6.8**±1.4
Gluconic acid, lactone	42.6±2.6	**28.9±1.7**	45.7±7.7	27.7±3.1	23.1±2.6	33.6±2.9
Glyceric acid	15.3±0.7	**24.6±1.3**	25.3±1.6	26.9±1.8	34.6±3.3	**21.2**±2.0
Glycolic acid	2.6±0.4	**5.2±0.1**	3.4±0.3	5.0±0.4	3.3±0.4	4.7±0.3
Gluonic acid	4.8±0.4	6.1±0.5	3.4±0.1	4.5±0.4	3.8±0.4	5.1±0.4
Hexanoic acid	0.3±0.1	0.3±0.0	0.2±0.1	0.4±0.1	0.1±0.1	0.2±0.0
Hydroxymalonic acid	0.2±0.0	**ND**	0.3±0.0	**ND**	0.2±0.0	ND
Lactic acid	139.8±10.7	**75.8±4.9**	179.9±41.0	112.0±7.0	273.4±36.3	**151.4**±35.9
Maleic acid	0.2±0.0	0.2±0.1	0.2±0.0	0.3±0.0	0.2±0.1	0.3±0.1
Malic acid	1056.2±83.1	**422.9±26.5**	1798.7±73.2	**528.5**±19.4	1891.3±94.9	**529.9**±38.6
Malonic acid	0.2±0.0	**0.9±0.2**	0.2±0.1	**0.7**±0.1	0.3±0.1	**1.9**±0.2
Nicotinic acid	6.8±1.0	4.7±0.8	6.0±0.3	4.1±0.2	3.9±0.6	3.7±0.4
Orotic acid	0.3±0.1	**ND**	0.2±0.0	**ND**	0.1±0.0	ND
Oxalic acid	5.9±0.6	6.4±1.0	10.2±1.2	**6.3**±0.3	5.2±0.6	6.1±0.5
Pyruvic acid	5.7±1.0	**9.7±0.3**	10.2±1.1	**21.5**±0.8	14.3±4.0	12.4±0.2
Quinic acid	2.6±0.1	**4.9±0.7**	6.2±1.3	7.7±0.3	12.1±2.9	14.0±2.3
Quinolinic acid	191.9±21.2	**3.5±0.4**	210.4±27.4	**4.9**±0.8	105.0±11.9	**10.7**±2.5
Ribonic acid	31.6±4.5	26.0±2.2	28.6±4.1	25.1±1.1	23.0±2.7	28.5±1.0
Shikimic acid	4.9±0.5	**2.8±0.3**	12.2±2.0	**2.4**±0.2	10.5±1.0	**1.6**±0.1
Succinic acid	77.2±12.4	**237.0±24.0**	74.5±3.6	**262.6**±28.8	124.3±29.2	**261.8**±19.6
Tartaric acid	2.9±0.3	4.5±0.2	6.0±1.5	3.8±0.1	5.2±0.3	7.0±0.8
Threonic acid	20.6±1.9	**37.6±0.6**	36.0±2.8	44.1±5.2	59.6±6.1	55.5±2.1
Threonic acid, lactone	1.1±0.3	1.3±0.3	1.0±0.2	1.5±0.2	1.1±0.3	1.3±0.2
Xylonic acid, lactone	10.9±2.0	**3.2±0.6**	10.0±1.4	**3.4**±0.3	6.5±0.5	**1.9**±0.2
Sterols						
Campesterol	3.2±0.4	**0.4**±0.1	3.8±0.1	**0.4**±0.1	2.7±0.3	**0.3**±0.1
Sitosterol	51.9±8.0	**26.4±0.4**	53.2±10.0	34.5±1.6	41.7±4.1	31.0±1.0

Tocopherol (Vitamine E)	4.2±0.7	**0.9±0.1**	4.2±0.7	**1.2**±0.0	3.0±0.7	**1.5**±0.3
Sugars						
1,6-Anhydroglucose	7.8±0.7	**3.7**±0.3	8.1±0.2	**2.6**±0.3	5.2±0.2	**2.6**±0.4
1-Ethylglucopyranoside	512.4±54.7	632.8±8.9	502.5±29.5	406.0±32.0	177.9±1.1	**381.4**±22.7
1-Methyl-α-D-galactopyranoside	3.4±0.3	**15.3±3.1**	2.9±0.1	**16.1**±3.6	5.8±1.4	**11.6**±1.2
1-Methyl-β-D-galactopyranoside	4.9±1.0	**28.5±3.5**	7.5±1.9	**60.6**±9.7	11.9±1.9	**39.2**±4.3
2-O-Glycerol-α-D-galactopyranoside	6.2±0.4	**31.4±4.8**	5.6±0.8	**34.8**±2.2	19.1±2.3	20.9±2.6
2-O-Glycerol-β-D-galactopyranoside	24.9±0.4	**67.2±2.9**	34.4±2.5	**69.4**±7.3	32.0±1.5	**70.2**±6.4
6-Deoxymannopyranose	4.1±0.3	**5.2±0.3**	3.4±0.2	3.8±0.5	1.9±0.1	**4.0**±0.2
Arabinose	3.8±0.2	4.6±0.3	14.4±1.4	**4.6**±0.8	15.2±0.1	**9.7**±0.4
Digalactosylglycerol	5.0±0.9	**20.8±0.6**	7.4±1.1	**49.2**±8.2	13.8±3.5	23.7±4.0
Fructofuranoside	857.3±21.3	**123.8±11.9**	614.8±35.0	**97.4**±13.1	481.9±49.5	**159.4**±25.8
Fructose	1083.5±257.7	**5257.7±361.8**	983.4±238.1	**5546.1**±261.6	1418.3±249.7	**6452.8**±275.1
Fructose-6-P	19.4±3.6	14.3±1.1	9.6±0.8	14.5±0.8	3.2±0.2	**0.5**±0.1
Galactofuranose	3.6±0.2	**19.0±2.8**	1.6±0.4	**15.1**±1.0	0.5±0.1	**20.2**±2.8
Galactosamine	ND	**2.2±0.2**	ND	**2.7**±0.3	ND	**2.8**±0.3
Galactose	223.3±39.8	**947.4±32.0**	240.5±36.2	**1301.0**±78.3	276.5±18.8	**1366.7**±95.0
Gentiobiose	91.3±5.7	**37.5±1.4**	80.8±4.4	**39.4**±8.8	74.1±3.0	**42.8**±2.4
Glucoheptulose	8.0±0.7	**10.6±1.6**	9.1±0.9	12.9±3.9	11.1±1.2	14.8±3.9
Glucopyranose	523.5±28.3	**2174.9±163.2**	663.7±41.7	**1636.3**±255.5	554.6±32.8	**1468.9**±248.3
Glucosamine	ND	**14.3±2.2**	ND	**10.6**±1.4	ND	**16.5**±3.0
Glucose	1211.0±163.7	**2720.8±199.0**	1251.8±141.9	**2948.8**±269.8	1447.5±78.0	**3567.0**±274.2
Glucose-1-P	19.7±3.4	24.9±2.5	22.9±1.9	34.8±0.9	9.4±1.2	**44.0**±4.1
Glucose-6-P	92.9±4.8	**23.0±0.9**	125.3±23.8	**24.3**±3.3	18.5±1.1	18.7±2.8
Isomaltose	3.1±0.5	3.0±0.3	19.1±3.8	**3.2**±0.4	16.9±2.3	**6.8**±0.6
Maltose	13.6±3.9	8.9±0.8	18.5±3.3	**7.8**±0.8	15.7±2.7	**7.1**±0.7
Mannose	4.1±0.8	**50.0±1.4**	47.7±4.4	50.0±2.8	51.6±2.1	58.3±4.3
Mannose-6-P	3.0±0.2	**1.4±0.1**	3.6±0.3	2.2±0.3	3.2±0.1	**1.9**±0.1
Melibiose	26.8±2.6	**12.5±1.1**	26.4±3.5	**14.3**±1.7	27.2±2.7	**11.0**±1.0
N-Acetyl glucosamine	1.8±0.5	1.4±0.2	1.8±0.1	1.6±0.2	1.5±0.4	1.6±0.2

N-Acetylglucosylamine	2.9±0.7	**ND**	2.4±0.3	**ND**	1.4±0.1	ND
Rhamnose	4.5±1.6	3.7±0.2	3.7±0.3	3.6±0.2	1.9±0.4	2.4±0.0
Ribose	11.8±1.5	**20.2±2.3**	15.7±2.5	**28.3**±3.7	25.7±4.4	15.3±2.2
Sedoheptulose	37.8±3.6	45.8±3.4	39.6±3.2	56.0±4.7	35.0±3.7	**62.3**±4.1
Sorbopyranose	488.5±45.0	**1716.4±183.3**	1034.8±163.7	**2034.0**±276.0	569.9±62.3	**1926.1**±180.0
Sorbose	812.5±51.3	**3246.0±224.6**	979.2±67.6	**3081.7**±47.0	1223.4±151.3	**3490.5**±293.1
Sucrose	4591.7±50.8	**2203.0±68.5**	4611.1±387.4	**2930.1**±107.2	4779.1±435.8	**3224.1**±447.2
Sucrose-6-P	2.5±0.6	1.7±0.2	3.2±0.7	**1.3**±0.1	0.5±0.1	**0.2**±0.0
Galactopyranose	1181.9±286.7	**2286.4±222.6**	1068.9±78.1	**2499.2**±173.6	966.6±28.6	**2525.6**±429.7
Trehalose	40.1±8.8	40.1±4.2	53.2±1.8	40.1±2.1	41.4±1.6	40.6±1.1
Trehalose-6-P	2.6±0.6	2.2±0.2	3.3±0.2	**1.6**±0.1	5.8±0.7	**1.9**±0.3
**Other metabolites**						
2,4,6-Tri-tert.-butylbenzenethiol	0.4±0.1	0.4±0.0	0.6±0.1	0.5±0.1	0.5±0.0	0.5±0.1
3,4-Hydroxy-2(3H)-Furanone	1.6±0.2	1.6±0.1	1.5±0.4	1.5±0.2	3.8±0.8	**1.5±0.2**
3-phosphoglycerate	6.1±0.2	3.6±0.5	3.1±0.4	2.7±0.2	0.3±0.1	**1.4±0.1**
β-Amyrin	0.5±0.1	0.4±0.1	1.3±0.3	1.5±0.1	1.1±0.2	1.7±0.3
Ethyleneglycol	1.7±0.4	1.9±0.3	1.4±0.2	**1.8±0.3**	1.5±0.2	**2.2±0.3**
Monomethylphosphate	1722.6±91.8	1611.2±93.9	1428.1±61.8	**1282.8±108.4**	346.5±29.7	**1354.0±216.2**
Pantothenic acid – Vitamin B5	0.4±0.1	**5.6±0.5**	1.5±0.5	**6.6±1.9**	0.2±0.0	**4.6±0.7**
3-[(2,2-dimethylpropylidene) amino]propylphosphate	30.8±2.3	45.6±4.2	13.8±0.4	**22.4±2.1**	5.1±0.3	**16.5±3.1**
Phosphate	3250.2±157.3	2856.1±138.7	2421.8±117.2	2412.2±291.4	990.2±71.7	**1483.9±330.8**
Ribofuranosyl-2(1H)-Pyrimidinone	5.0±0.4	2.7±0.8	7.5±1.4	**39.7±3.0**	22.1±1.2	**33.3±2.7**

Supplemental Figures **Page A.F. et al.**

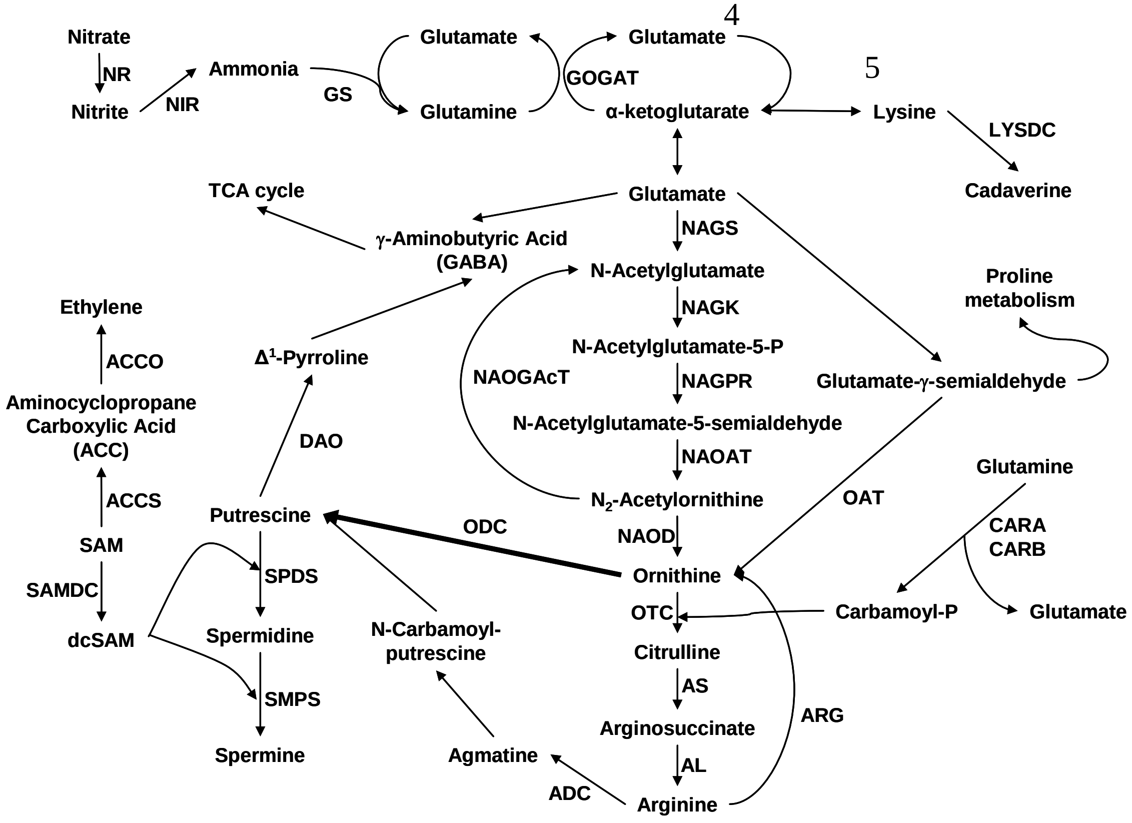

Supplemental Fig. S1. The pathway for the biosynthesis of polyamines and related metabolites starting from the assimilated nitrogen. ACC, 1-Aminocyclopropane-1-carboxylic acid oxidase; ADC, Arginine decarboxylase; AL, Argininosuccinate lyase; ARG, arginase; AS, Argininosuccinate synthase; CARA. Carbamoylphosphate synthase large subunit; CARB, small subunit of carbamoylphosphate synthase; DAO, Diamine oxidase; GAD, Glutamate decarboxylase; GOGAT, Glutamate synthase; GS, Glutamine synthetase; LysDC, Lysine decarboxylase; NAGK, N-Acetylglutamate kinase; NAGPR, N-Acetylglutamate-5-P reductase; NAGS, N-acetyl-glutamate synthase; NAOAT, N2-acetyl-Orn aminotransferase; NAOD, N2-acetyl-Orn deacetylase; NIR, Nitrite reductase; NR, Nitrate reductase; OAT, Ornithine aminotransferase; ODC, Ornithine decarboxylase; OTC, Ornithine transcarbamylase; SAMDC, S-adenosylmethionine decarboxylase;

SPDS, spermidine synthase; SMPS, spermine synthase. Native ODC (heavier arrow) is not expressed in control cells, but the transgenic mODC is over-expressed in HP cells.

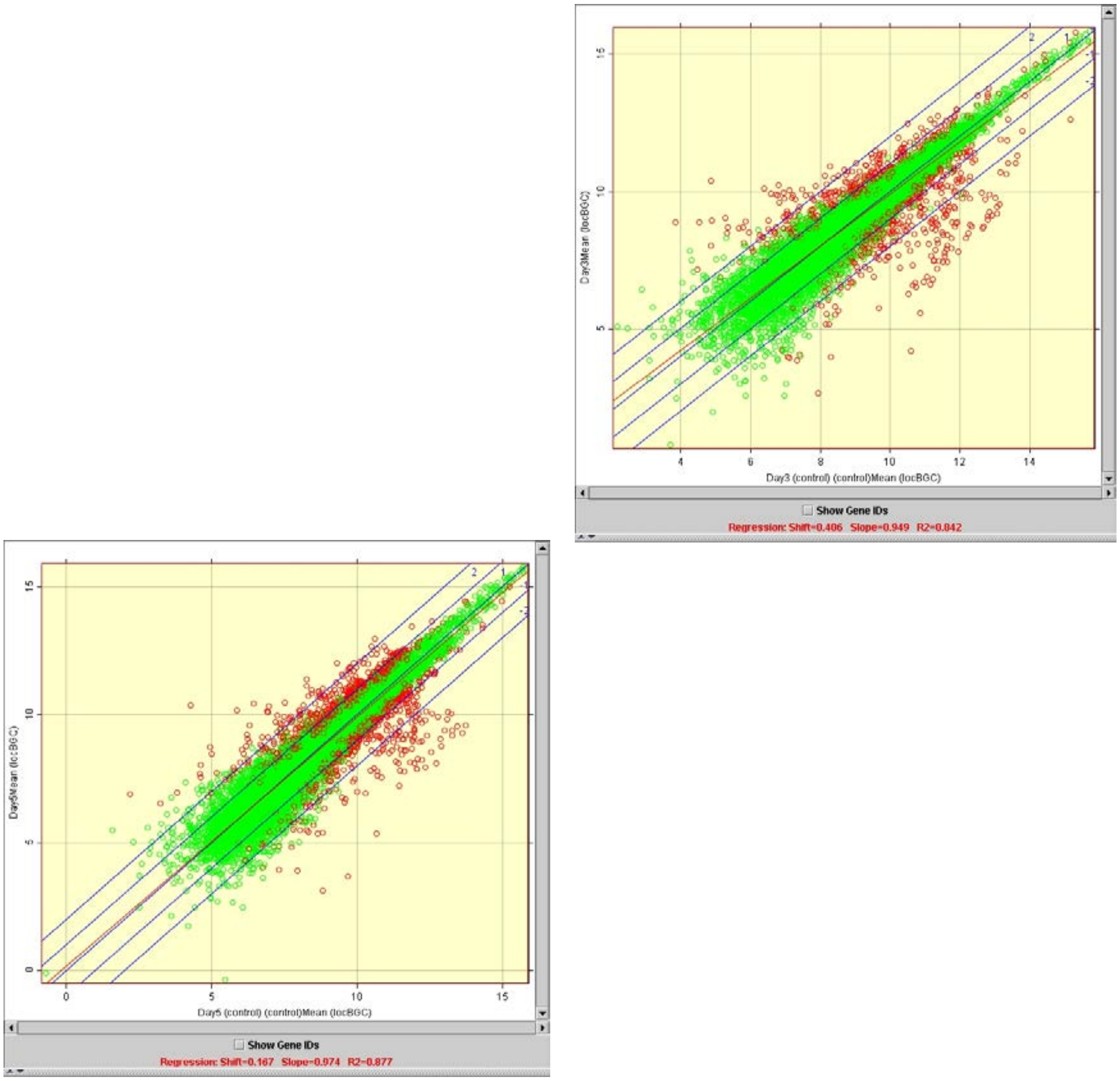

Supplemental Fig. S2. Quality control scatter plots showing expression levels of data that passed CV and dye-swap tests. Red spots indicate data that passed statistical analysis for differential expression between control and HP cell lines.

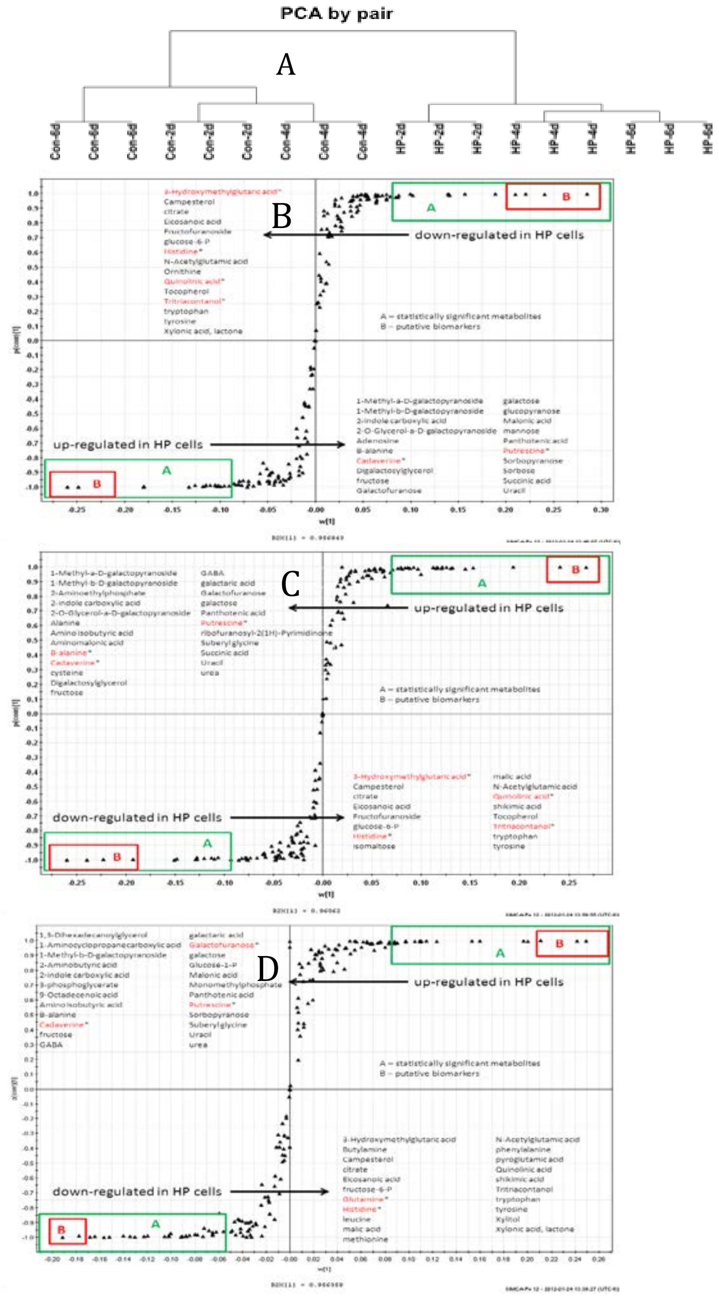

Supplemental Fig. S3. The loading plots (S-plot) of the OPLS-DA results for the control and HP cell extracts on days 2 (A), 4 (B), and 6 (C). In the S-plot, each point represents a single metabolite (marker). The *x*-axis shows the variable contributions. The farther away a data point is from the 0 value, the more it contributes to sample variance. The *y*-axis shows the sample correlations within the same sample group. The farther away a metabolite is from the 0 value, the better is its correlation from injection to injection. As a result, the metabolites on both ends of the S-shaped curve represent the leading contributing ions from each sample group. The OPLS-DA is a multivariate analysis model which separates the systematic variation in X into two parts, one that is linearly related (and therefore predictive) to Y and one that is orthogonal to Y (unrelated); the Y-predictive/related part represents the between-class variation, the Y-orthogonal (ToPo) part constitutes the within-class variation.
