# Supplementary material for: Genetic manipulation of putrescine biosynthesis reprograms the cellular transcriptome and the metabolome
Source: BMC Plant Biol. 2016 May 18;16:113. doi: 10.1186/s12870-016-0796-2 (PMC4870780; doi:10.1186/s12870-016-0796-2)
Supplement: Additional file 3: Table S1. — Summary of changes in expression of genes involved in polyamine metabolism. For abbreviations see Fig. 1. Sequences for AS, CARB, DAO, NAGK, NAGPR, NAOD, NAOGAcT, ODC, OTC and SPDS were not represented on the microarray. Table S2. Functional clustering of gene models showing significant (P ≤ 0.05) differences (≥2 fold) between the HP and the control cell lines on both day 3 and day 5. Table S3. Functional clustering of gene models showing significant (P ≤ 0.05) differences (≥2 fold) between the HP and the control cell lines on day 3 only. Table S4. Functional clustering of gene models showing significant (P ≤ 0.05) differences (≥2 fold) between the HP and the control cell lines on day 5 only. Table S5. List of metabolites that were positively identified in poplar control and HP cell lines. ND = not detectable. Values that are significantly different (P ≤ 0.05) in the HP cells from the corresponding control cells on a given day are marked in bold. (DOCX 103 kb) [file 12870_2016_796_MOESM3_ESM.docx]

## **Authors: Page A.F. et al.**

**Additional file 3: TABLES**

**Additional file Table S1:**

**Additional file Table S2.**

**Additional file Table S3.**

**Additional file Table S4.**

**Additional file Table S5:**

**Additional file 3: Table S1:** Summary of changes in expression of genes involved in polyamine metabolism. For abbreviations see Figure S1. Sequences for AS, CARB, DAO, NAGK, NAGPR, NAOD, NAOGAcT, ODC, OTC and SPDS were not represented on the microarray. For original data, see: [geo@ncbi.nlm.nih.gov](mailto:geo@ncbi.nlm.nih.gov) - Number still awaited – see attached email.

| Gene name | Spots | Gene models | **3 d** | | **5 d** | | **3 and 5 d** | |
| --- | --- | --- | --- | --- | --- | --- | --- | --- |
|  |  |  | Spots passing CV + dye swap | Gene models passing CV + dye swap | Spots passing CV + dye swap | Gene models passing CV + dye swap | Spots passing CV + dye swap | Gene models passing CV + dye swap |
| ACCO | 13 | 6 | 3 | 2 | 8 | 3 | 2 | 2 |
| ACCS | 10 | 7 | 6 | 4 | 9 | 6 | 6 | 4 |
| AL | 1 | 1 | 0 | 0 | 1 | 1 | 0 | 0 |
| ARG | 2 | 1 | 0 | 0 | 1 | 1 | 0 | 0 |
| ADC | 6 | 1 | 1 | 1 | 4 | 1 | 1 | 1 |
| CARA | 1 | 1 | 0 | 0 | 1 | 1 | 0 | 0 |
| GAD | 5 | 1 | 1 | 1 | 3 | 1 | 0 | 0 |
| GOGAT | 4 | 3 | 1 | 1 | 2 | 2 | 1 | 1 |
| GS | 28 | 7 | 12 | 4 | 21 | 6 | 11 | 4 |
| LYSDC | 5 | 5 | 0 | 0 | 1 | 1 | 0 | 0 |
| NAGS | 2 | 2 | 1 | 1 | 1 | 1 | 1 | 1 |
| NAOAT | 3 | 2 | 1 | 1 | 1 | 1 | 0 | 0 |
| NAOD | 0 | 0 | 0 | 0 | 0 | 0 | 0 | 0 |
| NR | 2 | 1 | 2 | 1 | 2 | 1 | 2 | 1 |
| NiR | 3 | 1 | 0 | 0 | 1 | 1 | 0 | 0 |
| OAT | 3 | 2 | 1 | 1 | 0 | 0 | 0 | 0 |
| SAMDC | 15 | 4 | 7 | 4 | 7 | 2 | 3 | 1 |
| SPMS | 3 | 2 | 1 | 1 | 0 | 0 | 0 | 0 |

**___________________________________________________________________________________________________________**

**Additional file 3: Table S2.** Functional clustering of gene models showing significant (p<0.05) differences (>2 fold) between the HP and the control cell lines on both day 3 and day 5. **The specific Gene Ontology numbers associated with Molecular function/Biological process were obtained by searching with the gene names for Gene Ontology terms associated with plants using EMBL Quick go (**[**http://www.ebi.ac.uk/QuickGO/**](http://www.ebi.ac.uk/QuickGO/)**). Bold** = up regulated; normal = down regulated. For original data, see: [geo@ncbi.nlm.nih.gov](mailto:geo@ncbi.nlm.nih.gov) - Number still awaited – attached email.

|  | **Specific GO number/s** | **Molecular function (MF) and/or Biological process (BP)** | **3 d Fold Change** | **5 d Fold Change** | **# ESTs for this model** |
| --- | --- | --- | --- | --- | --- |
| **Enzymatic** | | | | | |
| Glutathione-s-transferase | GO:0016740 | MF: transferase activity | **2.87** | **2.21** | 1 |
| Acetyltransferase | GO:2000983 | MF: regulation of acetyl-CoA:oxaloacetate acetyltransferase (isomerizing; ADP- phosphorylating) activity | **2.66** | **3.75** | 1 |
| Alcohol dehydrogenase 2 | GO:0052933,GO:0052934 | MF: alcohol dehydrogenase [cytochrome c(L)] activity, MF: alcohol dehydrogenase [cytochrome c] activity | -2.07 | -2.31 | 1 |
| Ubiquitin-conjugating enzyme E2-17 (UBC9) | GO:0035370 | MF: catalyzes assembly of linked polyubiquitin chains | -2.39 | -2.03 | 1 |
| Cobalamine-independent methionine synthase | GO:0008705 | MF:methionine synthase activity | -2.88 | -2.69 | 1 |
| Pectate lyase | GO:0030570 | MF:pectic acid transeliminase activity and/or pectic acid lyase activity | -3.23 | -2.53 | 4 |
| Dolichyl-di-phosphooligosaccharide glycotransferase | GO:0004579 | MF: dolichyl-diphosphooligosaccharide:protein-L-asparagine oligopolysaccharidotransferase activity | -5.94 | -2.27 | 1 |
| **Ribosomal/transcription/translation** | | | | | |
| Small nuclear ribonucleoprotein-like protein | GO:0006397 | BP: Any process involved in the conversion of a primary mRNA transcript into one or more mature mRNA(s) prior to translation into polypeptide. mRNA maturation | **3.83** | **2.85** | 1 |
| F2D10.18 transcription regulator | GO:0001068 | MF: Interacting selectively and non-covalently with a DNA region that regulates the transcription of a region of DNA | **3.68** | **2.04** | 1 |
| Histone H2A | GO:0043968,GO:0033522,GO:1990164,  GO:0035518 | BP: histoneH2A acetylation; histoneH2A ubiquitination; histoneH2A phosphorylation; histoneH2A monoubiquitination | **2.6** | **2.03** | 2 |
| Poly(A)-binding protein | GO:0003723, GO:0000398 | MF: RNA binding, BP: nuclear mRNA splicing, via spliceosome | -2.16 | -2.58 | 1 |
| Translation initiation regulator like | GO:0031369 | MF: polypeptide factor involved in the initiation of ribosome-mediated translation | -5.15 | -4.29 | 1 |
| **Membrane transport and osmoregulation** | | | | | |
| Plasma membrane intrinsic protein | GO:0005215, GO:0006810 | MF: transporter activity, BP: transport | **9.95** | **7.12** | 6 |
| Aquaporin TIP3 | GO:0015288, GO:0005215, GO:0006810 | MF: porin activity, MF: transporter activity, BP: transport | -2.77 | -2.24 | 1 |
| Annexin Anx1 | GO:0005509, GO:0005544 | MF: calcium ion binding, MF: calcium-dependent phospholipid binding | -3.79 | -2.36 | 1 |
| **Stress/Wound associated** | | | | | |
| Wound induced protein | GO:0009611 | BP: response to wounding | **7.84** | **4.09** | 2 |
| Basic chitinase | GO:0004568 | MF:Catalysis of the hydrolysis of (1->4)-beta linkages of N-acetyl-D-glucosamine (GlcNAc) polymers of chitin and chitodextrins | **4.05** | **5.7** | 2 |
| Chitinase | GO:0004568 | MF:Catalysis of the hydrolysis of (1->4)-beta linkages of N-acetyl-D-glucosamine (GlcNAc) polymers of chitin and chitodextrins | **3.11** | **7.14** | 1 |
| HEV1.2, hevein | GO:0008061 | MF: chitin binding | **2.83** | **3.95** | 1 |
| Metallothionein 2a | GO:0046872 | MF: Metal ion binding | **2.57** | **4.09** | 2 |
| Heat shock protein | GO:0031072 | MF: Interacting selectively and non-covalently with a heat shock protein, any protein synthesized or activated in response to heat shock | **2.52** | **2.17** | 1 |
| Metallothionein 2b | GO:0046872 | MF: Metal ion binding | **2.18** | **2.44** | 1 |
| Small heat shock protein - soybean | GO:0031072 | MF: Interacting selectively and non-covalently with a heat shock protein, any protein synthesized or activated in response to heat shock | **1.01** | -1.05 | 2 |
| Ascorbate peroxidase | GO:0004601, GO:0006979 | MF: peroxidase activity, BP: response to oxidative stress | -2.21 | -2.18 | 1 |
| Pathogenesis-related protein | GO:0006952 | MF: Biological process: defense response | -2.45 | -3.77 | 1 |
| Stress related protein | GO:0006950 | BP: response to any kind of stress | -2.51 | -2.31 | 1 |
| Cationic peroxidase | GO:0005506, GO:0016491,GO:0004601, GO:0006979 | MF: iron ion binding, MF: oxidoreductase activity, MF: peroxidase activity, BP: response to oxidative stress | -5.39 | -4.07 | 17 |
| **Cell wall** | | | | | |
| Cell wall-plasma membrane linker protein | GO:0005199 | MF: structural constituent of cell wall | -2.04 | -2.29 | 1 |
| 3,5-epimerase/4-reductase | GO:0003824,GO:0008831,GO:0051287 | MF: catalytic activity, MF: dTDP-4-dehydrorhamnose reductase activity, MF: NAD binding | -3.29 | -2.25 | 1 |
| Fasciclin-like AGP 10 | GO:0007155 | BP: cell adhesion | -4.84 | -4.7 | 1 |
| Extensin like protein - black poplar | GO:0010409 | BP: extensin metabolic process | -5.87 | -5.81 | 5 |
| Extensin like protein - black poplar | GO:0010409 | BP: extensin metabolic process | -8 | -8.46 | 8 |
| Extensin like protein | GO:0010409 | BP: extensin metabolic process | -16.28 | -18.46 | 12 |

**Additional file 3: Table S3.** Functional clustering of gene models showing significant (p<0.05) differences (>2 fold) between the HP and the control cell lines on day 3 only. **Bold** = up regulated; normal = down regulated. For original data, see: [geo@ncbi.nlm.nih.gov](mailto:geo@ncbi.nlm.nih.gov) - Number still awaited – see attached email.

|  | **Specific GO number/s** | **Molecular function and/or biological process** | **Fold Change** | **# ESTs for this model** |
| --- | --- | --- | --- | --- |
| **Enzymatic** | | | | |
| NADH dehydrogenase subunit 1 | GO:0003954 | MF: NADH2 dehydrogenase activity | **3.29** | 1 |
| Glyceraldehyde-3-phosphate dehydrogenase | GO:0008943 | MF: Glyceraldehyde-3-phosphate dehydrogenase activity | **3.25** | 1 |
| Ubiquinol--cytochrome c reductase | GO:0008121 | MF: Catalysis of the transfer of a solute or solutes from one side of a membrane to the other according to the reaction | **2.61** | 1 |
| Beta-amylase | GO:0016161, GO:0000272 | MF: beta-amylase activity; BP: polysaccharide catabolism | **2.37** | 1 |
| Fructose-bisphosphate aldolase | GO:0004332, GO:0006096 | MF: fructose-bisphosphate aldolase activity; BP: glycolysis | -2.07 | 3 |
| Phenylcoumaran benzylic ether reductase | GO:0032442 | MF: Catalysis of the NADPH-dependent 7-O-4' reduction of phenylcoumaran lignans to the corresponding diphenols | -2.15 | 1 |
| Serine peptidase | GO:0008236 | MF: Catalysis of the hydrolysis of peptide bonds in a polypeptide chain by a catalytic mechanism that involves a catalytic triad consisting of a serine nucleophile that is activated by a proton relay involving an acidic residue (e.g. aspartate or glutamate) and a basic residue (usually histidine). | -2.17 | 1 |
| Peptidyl-prolyl cis-trans isomerase | GO:0003755 | MF: Peptidyl-prolyl cis-trans isomerase activity | -2.18 | 2 |
| RUB1 conjugating enzyme | GO:0008642, GO:0006512 | MF: ubiquitin-like activating enzyme activity, BP: ubiquitin cycle | -2.24 | 2 |
| Methionine synthase | GO:0008168, GO:0009086 | MF: methyltransferase activity, BP: methionine biosynthesis | -2.25 | 1 |
| Photosystem II 32 kDa protein | GO:0019684 | BP:photosynthesis, light reaction | -2.28 | 1 |
| Glucose-6-phosphate dehydrogenase | GO:0004345 | MF: glucose-6-phosphate 1-dehydrogenase activity | -2.37 | 1 |
| Pectate lyase | GO:0030570 | MF:pectic acid transeliminase activity and/or pectic acid lyase activity | -2.39 | 1 |
| Polygalacturonase-like protein | GO:0004650 | MF:random hydrolysis of (1->4)-alpha-D-galactosiduronic linkages in pectate and other galacturonans | -2.43 | 1 |
| Ubiquitin-conjugating enzyme 9 | GO:0035370 | MF: catalyzes assembly of linked polyubiquitin chains | -2.63 | 3 |
| Aspartic proteinase | GO:0070001 | MF: aspartic-type peptidase activity | -2.74 | 2 |
| Cytosolic phosphoglucomutase | GO:0004614 | MF:Catalysis of the conversion reaction: alpha-D-glucose 1-phosphate = alpha-D-glucose 6-phosphate | -3.19 | 1 |
| Formate dehydrogenase | GO:0008863 | MF: formate dehydrogenase (NAD+) activity | -3.31 | 1 |
| P protein, component of aminomethyltransferase | GO:0004047 | MF:aminomethyltransferase activity | -3.63 | 1 |
| Malate dehydrogenase | GO:0016615 | MF: Catalysis of the reversible conversion of pyruvate or oxaloacetate to malate | -3.85 | 1 |
| Wax synthase isoform 1 | GO:0047196, GO:0016740 | MF: long-chain-alcohol O-fatty-acyltransferase activity, MF: transferase activity | -3.86 | 1 |
| Pectate lyase | GO:0030570 | MF: pectin transeliminase activity or pectic acid lyase activity | -4.45 | 1 |
| **Ribosomal/transcription/translation** | | | | |
| Acidic ribosomal protein P1a | GO:0003735, GO:0006610 | MF:contributes to the structural integrity of the ribosome, BP: ribosomal protein import into nucleus | **3.15** | 1 |
| Ribosomal protein L9 | GO:0003735, GO:0006610 | MF:contributes to the structural integrity of the ribosome, BP: ribosomal protein import into nucleus | **3.1** | 1 |
| Histone H2A | GO:0043968, GO:0033522, GO:1990164, GO:0035518 | BP: histoneH2A acetylation; histoneH2A ubiquitination; histoneH2A phosphorylation; histoneH2A monoubiquitination | **2.92** | 2 |
| Histone H1 | GO:0018024 | MF: histone H1-specific S-adenosylmethionine:protein-lysine N-methyltransferase activity | **2.38** | 1 |
| Acidic ribosomal protein P1a | GO:0003735, GO:0006610 | MF:contributes to the structural integrity of the ribosome, BP: ribosomal protein import into nucleus | **2.15** | 1 |
| Ribosomal protein | GO:0003735, GO:0006610 | MF:contributes to the structural integrity of the ribosome, BP: ribosomal protein import into nucleus | **2.14** | 1 |
| Ribosomal protein S19 | GO:0003735, GO:0006610 | MF:contributes to the structural integrity of the ribosome, BP: ribosomal protein import into nucleus | **2.02** | 1 |
| Ribosomal protein S4 | GO:0003735, GO:0006610 | MF:contributes to the structural integrity of the ribosome, BP: ribosomal protein import into nucleus | -2.07 | 1 |
| Ribosomal protein L7A | GO:0003735, GO:0006610 | MF:contributes to the structural integrity of the ribosome, BP: ribosomal protein import into nucleus | -2.17 | 1 |
| Histone protein 60 | - | - | -2.28 | 1 |
| Ribosomal protein L10 | GO:0003735, GO:0006610 | MF:contributes to the structural integrity of the ribosome, BP: ribosomal protein import into nucleus | -4.24 | 1 |
| Transcription factor-like | GO:0001070 | MF:Interacting selectively and non-covalently with an RNA sequence to modulate transcription | -9.36 | 1 |
| **Membrane transport and osmoregulation** | | | | |
| Plasma membrane intrinsic protein | GO:0005215, GO:0006810 | MF: transporter activity, BP: transport | **10.08** | 1 |
| Hydrogen-transporting ATP synthase activity | GO:0046933 | MF: hydrogen ion transporting two-sector ATPase activity | **2.39** | 1 |
| Plasma membrane intrinsic protein | GO:0005215, GO:0006810 | MF: transporter activity, BP: transport | **2.27** | 1 |
| Plasma membrane intrinsic protein | GO:0005215, GO:0006810 | MF: transporter activity, BP: transport | -2.06 | 1 |
| Annexin | GO:0005509, GO:0005544 | MF: calcium ion binding, MF: calcium-dependent phospholipid binding | -2.88 | 2 |
| Aquaporin | GO:0015288, GO:0005215 | MF: porin activity, MF: transporter activity | -3.08 | 1 |
| Osmotin | GO:0006970 | BP: osmotic stress response | -5.46 | 2 |
| **Stress/Wound associated** | | | | |
| Heat shock protein | GO:0031072 | MF: protein synthesized or activated in response to heat shock | **3.85** | 1 |
| Cytosolic class II low MW heat shock protein | GO:0031073 | MF: protein synthesized or activated in response to heat shock | **2.86** | 1 |
| Heat shock protein | GO:0031072 | MF: protein synthesized or activated in response to heat shock | **2.29** | 1 |
| Proteinase inhibitor se60-like protein (defence) | GO:1900277 | BP: Any process that stops, prevents or reduces the frequency, rate or extent of proteinase activated receptor activity | -2.06 | 1 |
| Dehydration stress-induced protein | GO:0009414 | BP: response to water deprivation | -2.06 | 1 |
| Ascorbate peroxidase | GO:0004601, GO:0006979 | MF: peroxidase activity, BP: response to oxidative stress | -2.13 | 1 |
| Cationic peroxidase 2 | GO:0005506, GO:0016491, GO:0004601, GO:0006979 | MF: iron ion binding, MF: oxidoreductase activity, MF: peroxidase activity, BP: response to oxidative stress | -2.2 | 1 |
| Pathogenesis-related protein | GO:0006952 | BP: defense response | -3.97 | 1 |
| **Cell wall** | | | | |
| 3,5-epimerase/4-reductase | GO:0003824, GO:0008831, GO:0051287 | MF: catalytic activity, MF: dTDP-4-dehydrorhamnose reductase activity, MF: NAD binding | -2.83 | 1 |

**Additional file 3: Table S4.** Functional clustering of gene models showing significant (p<0.05) differences (>2 fold) between the HP and the control cell lines on day 5 only. **Bold** = up regulated; normal = down regulated. For original data, see: [geo@ncbi.nlm.nih.gov](mailto:geo@ncbi.nlm.nih.gov) - Number still awaited – see attached email.

|  | | **Specific GO number/s** | **Molecular function and/or biological process** | **Fold Change** | **# ESTs this model** |
| --- | --- | --- | --- | --- | --- |
| **Enzymatic** | | | | | |
| NADH dehydrogenase ubiquinone | GO:0008137 | | MF: Catalysis of the reaction: NADH + H+ + ubiquinone = NAD+ + ubiquinol | **3.87** | 2 |
| Tropinone reductase | GO:0050358 | | MF: tropinone (psi-tropine-forming) reductase activity | **2.96** | 3 |
| Gamma-glutamylcysteine synthetase | GO:0004357 | | gamma-glutamyl-L-cysteine synthetase activity | **2.71** | 1 |
| Alternative oxidase | GO:0009916, GO:0010230 | | MF: alternative oxidase activity, BP: alternative respiration | **2.68** | 1 |
| Cinnamate 4-hydroxylase | GO:0016710 | | MF: t-cinnamic acid hydroxylase activity, Catalysis of the reaction: trans-cinnamate + NADPH + H+ + O2 = 4-hydroxycinnamate + NADP+ + H2O | **2.35** | 1 |
| Glycerophosphoryl diester phosphodiesterase family protein | GO:0008889 | | MF:glycerophosphoryl diester phosphodiesterase activity Catalysis of the reaction: a glycerophosphodiester + H2O = an alcohol + sn-glycerol 3-phosphate | **2.12** | 1 |
| E2, ubiquitin-conjugating enzyme | GO:0008642, GO:0006512, GO:0032355 | | MF: ubiquitin-like activating enzyme activity, BP: ubiquitin cycle, BP: Cellular response to estradiol stimulus | **2.08** | 1 |
| Laccase | GO:0005507, GO:0016491, GO:0065009 | | MF: copper ion binding, MF: oxidoreductase activity, BP: regulation of molecular function | -2.01 | 1 |
| UDP-glucoronosyl/UDP-glucosyl transferase family protein | GO:0035251, GO:0097359 | | MF:Catalysis of the transfer of a glucosyl group from UDP-glucose to an acceptor molecule, BP:UDP-glucosylation | -2.05 | 1 |
| Acyl-CoA independent ceramide synthase | GO:0097001, GO:0006672 | | MF:ceramide binding, BP:ceramide metabolic process and pathways involving ceramides | -2.18 | 1 |
| Chalcone isomerase | GO:0045430, GO:0009714 | | MF:Catalysis of the isomerase reaction: a chalcone = a flavanone, chalcone metabolic process | -2.32 | 1 |
| Adenosylhomocysteinase | GO:0004013, GO:0006730 | | MF: adenosylhomocysteinase activity, BP: one-carbon compound metabolism | -2.44 | 1 |
| NADPH-cytochrome P450 oxydoreductase isoform 3 | GO:0015034 | | MF: Cytochrome P450 activity | -2.9 | 1 |
| **Ribosomal/transcription/translation** | | | | | |
| Histone H2A | | GO:0043968, GO:0033522, GO:1990164, GO:0035518 | BP: histoneH2A acetylation; histoneH2A ubiquitination; histoneH2A phosphorylation; histoneH2A monoubiquitination | 2.44 | 1 |
| DNA-binding protein | | GO:0003677, GO:0008642, GO:0006512 | MF: DNA binding, MF: ubiquitin-like activating enzyme activity, BP: ubiquitin cycle | 2.06 | 1 |
| Ribosomal protein L34 | | GO:0003735, GO:0006610 | MF:contributes to the structural integrity of the ribosome, BP: ribosomal protein import into nucleus | -2.04 | 1 |
| Ribosomal protein L30 | | GO:0003735, GO:0006610 | MF:contributes to the structural integrity of the ribosome, BP: ribosomal protein import into nucleus | -2.08 | 1 |
| Ribosomal protein S25 | | GO:0003735, GO:0006610 | MF:contributes to the structural integrity of the ribosome, BP: ribosomal protein import into nucleus | -2.12 | 2 |
| Ribosomal protein S4 | | GO:0003735, GO:0006610 | MF:contributes to the structural integrity of the ribosome, BP: ribosomal protein import into nucleus | -2.15 | 1 |
| Nucleolar protein | | GO:0034503 | BP: Process in which a protein is transported to, or maintained at, the rDNA repeats on a chromosome in the nucleolus | -2.21 | 1 |
| Ribosomal protein L17-1 | | GO:0003735, GO:0006610 | MF:contributes to the structural integrity of the ribosome, BP: ribosomal protein import into nucleus | -2.22 | 1 |
| Ribosomal protein S11 | | GO:0003735, GO:0006610 | MF:contributes to the structural integrity of the ribosome, BP: ribosomal protein import into nucleus | -2.22 | 1 |
| Ribosomal protein S11 | | GO:0003735, GO:0006610 | MF:contributes to the structural integrity of the ribosome, BP: ribosomal protein import into nucleus | -2.24 | 2 |
| Eukaryotic translation initiation factor 2 alpha subunit eIF2 | | GO:0003743 | MF: functions in the initiation of ribosome-mediated translation of mRNA into a polypeptide | -2.31 | 1 |
| Ribosomal protein S30 | | GO:0003735, GO:0006412 | MF: structural constituent of ribosome, BP: protein biosynthesis | -2.43 | 2 |
| Transcription factor LIM | | GO:0001070 | MF: Interacting selectively and non-covalently with RNA in order to modulate transcription. | -2.46 | 1 |
| **Membrane transport and osmoregulation** | | | | | |
| Imbibition protein | | - | - | **2.39** | 1 |
| Aquaporin | | GO:0015288, GO:0005215 | MF: porin activity, MF: transporter activity | **2.25** | 1 |
| Plasma membrane intrinsic protein | | GO:0005215, GO:0006810 | MF: transporter activity, BP: transport | **2.23** | 1 |
| Plasma membrane intrinsic protein | | GO:0005215, GO:0006810 | MF: transporter activity, BP: transport | **2.05** | 1 |
| Vacuolar V-H+ATPase subunit E | | GO:0070072 | BP: proton-transporting two-sector ATPase complex that couples ATP hydrolysis to the transport of protons across the vacuolar membrane | **2.04** | 1 |
| Vacuolar V-H+ATPase subunit E | | GO:0070072 | BP: proton-transporting two-sector ATPase complex that couples ATP hydrolysis to the transport of protons across the vacuolar membrane | **2.04** | 1 |
| Vacuole-associated annexin VCaB42 | | GO:0005509 | MF: calcium ion binding ,calcium-dependent phospholipid binding | -2.05 | 1 |
| **Stress/Wound associated** | | | | | |
| Peroxidase ATPA2 | | GO:0005506, GO:0016491, GO:0004601, GO:0006979 | MF: iron ion binding, MF: oxidoreductase activity, MF: peroxidase activity, BP: response to oxidative stress | **2.5** | 1 |
| Metallothionein 2b | | GO:0046872 | MF: Metal ion binding | **2.35** | 3 |
| Beta-1,3-glucanase | | GO:0033903 | MF: endo-1,3(4)-beta-glucanase activity | **2.3** | 1 |
| Metallothionein 1b | | GO:0046872 | MF: Metal ion binding | **2.13** | 26 |
| Metallothionein 1a | | GO:0046872 | MF: Metal ion binding | **2.11** | 2 |
| Metallothionein 1a | | GO:0046872 | MF: Metal ion binding | **2.1** | 1 |
| Metallothionein 2a | | GO:0046872 | MF: Metal ion binding | **2.06** | 1 |
| Cold stress protein SRC1 | | GO:0009409 | BP:response to cold stress | **2.05** | 1 |
| Metallothionein 1a | | GO:0046872 | MF: Metal ion binding | **2.02** | 1 |
| SCOF-1 | | - | - | -3.13 | 1 |
| **Cell Wall** | | | | | |
| 3,5-epimerase/4-reductase | | GO:0003824, GO:0008831, GO:0051287 | MF: catalytic activity, MF: dTDP-4-dehydrorhamnose reductase activity, MF: NAD binding | -2.36 | 1 |
| Xyloglucan endotransglycosylase | | GO:0016762 | MF: xyloglucan endotransglycosylase activity | -2.71 | 2 |

**Additional file 3: Table S5:** List of metabolites that were positively identified in poplar control and HP cell lines. ND = not detectable. GC/MS detected a total of 645 compounds in the control cell line and 680 in the HP cell line. Of these, 190 and 178 compounds were positively identified in the control and the HP cells, respectively with the total number of positively identified compounds being >200. Values that are significantly different (P < 0.05) in the HP cells from the corresponding control cells on a given day are marked in **bold**. For original data, see: <https://mynotebook.labarchives.com/share/ulav72/MjIuMXwxNzEzMTkvMTcvVHJlZU5vZGUvMzg1Mzg2MTkxNHw1Ni4x>.

| **Relative conc. g FW^-1^** | **Con-2d** | **HP-2d** | **Con-4d** | **HP-4d** | **Con-6d** | **HP-6d** |
| --- | --- | --- | --- | --- | --- | --- |
| ***Alcohols*** | | | | | | |
| Erythritol | 7.6±1.2 | **4.1±0.7** | 6.8±0.3 | 7.1±0.8 | 6.5±0.6 | 7.1±0.5 |
| Galactitol | 2.9±0.2 | **ND** | 1.4±0.2 | **ND** | ND | ND |
| Glycerol | 182.2±12.8 | **135.0±3.9** | 154.1±20.9 | 134.6±18.0 | 119.6±10.2 | 188.2±19 |
| Glycerol-2-P | 0.7±0.1 | **ND** | 1.7±0.2 | **ND** | 0.4±0.1 | **ND** |
| Glycerol-3-p | 17.3±4.1 | 14.7±3.1 | 11.1±1.5 | 13.7±4.3 | 4.3±0.4 | 8.3±1.5 |
| Inositol | 2247.2±199.6 | 2601.0±280.1 | 3288.8±251.3 | 3133.5±95.1 | 3237.0±262.0 | 3718.6±97.5 |
| Inositol-P | 30.5±2.9 | **16.0±3.5** | 33.7±3.3 | **12.8**±1.2 | 26.6±3.5 | 25.4±3.5 |
| Maltitol | 1.0±0.2 | **ND** | 1.1±0.2 | **ND** | ND | ND |
| Mannitol | 3.3±0.2 | 3.4±0.4 | 3.8±0.3 | 3.4±0.3 | 5.0±0.7 | 4.9±0.3 |
| Octadecanol | 1.0±0.1 | **0.7±0.1** | 1.2±0.2 | 1.1±0.2 | 1.2±0.3 | 1.0±0.1 |
| Ribitol | 3.1±0.8 | **9.5±0.6** | 8.5±1.2 | 6.0±0.4 | 9.3±0.8 | 6.7±1.5 |
| Sorbitol | 7.0±1.2 | **4.1±0.7** | 6.2±2.2 | 6.4±1.3 | 6.4±2.9 | 2.6±0.3 |
| Sorbitol-6-P | 5.2±0.8 | **3.1±0.1** | 7.4±1.5 | 3.8±0.3 | 7.1±0.8 | **3.9**±0.3 |
| Tetracosanol | 0.3±0.1 | 0.2±0.0 | ND | **0.2**±0.1 | ND | ND |
| Tetratriacontanol | 1.2±0.2 | 1.2±0.2 | 1.3±0.2 | 1.5±0.3 | 1.3±0.3 | 1.2±0.4 |
| Threitol | 1.4±0.4 | **3.3±0.5** | 3.8±0.6 | 2.7±0.6 | 3.6±0.5 | 3.0±0.5 |
| Triacontanol | 0.4±0.1 | 0.5±0.1 | 0.2±0.2 | 0.5±0.1 | 0.5±0.1 | 0.5±0.0 |
| Tritriacontanol | 8.7±1.9 | **0.4±0.1** | 10.1±2.6 | **0.3**±0.0 | 6.1±0.6 | **0.5**±0.1 |
| Xylitol | 3.7±0.5 | 3.4±0.3 | 9.3±1.4 | **3.8**±0.7 | 10.5±1.3 | **2.3**±0.3 |
| ***Lipids*** | | | | | | |
| 1,3-Dihexadecanoylglycerol | 0.7±0.2 | **1.4±0.2** | 0.9±0.1 | **2.7**±0.4 | 0.8±0.1 | **3.0**±0.3 |
| 11,14,17-Eicosatrienoic acid | 2.8±0.1 | 2.9±0.2 | 3.9±0.2 | 3.0±0.4 | 3.4±0.3 | 2.3±0.3 |
| 1-Monohexadecanoylglycerol | 8.1±0.5 | **2.8±0.2** | 4.0±0.7 | 5.4±1.2 | 3.0±0.4 | **5.9**±0.9 |
| 9,12,15-Octadecatrienoic acid | 56.1±2.2 | 49.5±2.2 | 74.4±6.3 | 101.6±13.9 | 76.4±2.4 | 85.8±4.7 |
| 9,12-Octadecadienoic acid | 75.6±3.5 | **28.3±3.6** | 63.2±2.1 | **34.3**±2.7 | 66.8±4.4 | 52.6±8.3 |
| 9-Octadecenoic acid | 12.0±2.3 | **3.9±0.1** | 5.5±1.3 | **13.7**±2.5 | 1.5±0.5 | **24.6**±1.4 |
| Docosanoic acid | 2.9±0.2 | **1.4±0.1** | 3.1±0.2 | **1.9**±0.3 | 2.9±0.1 | **1.8**±0.1 |
| Dodecanoic acid | 1.2±0.1 | 1.4±0.2 | 1.4±0.4 | 1.7±0.3 | 1.7±0.5 | 1.5±0.3 |
| Eicosanoic acid | 1.3±0.3 | **0.1±0.0** | 1.2±0.4 | **0.1**±0.0 | 1.3±0.4 | **0.1**±0.0 |
| Heptadecanoic acid | 1.9±0.4 | 1.8±0.3 | 1.6±0.3 | 1.7±0.3 | 1.9±0.2 | 1.5±0.3 |
| Heptanoic acid | 0.6±0.1 | 0.7±0.1 | 0.6±0.1 | 0.7±0.1 | 0.5±0.1 | 0.7±0.0 |
| Hexacosanoic acid | 0.5±0.1 | 0.6±0.1 | 0.5±0.1 | 0.5±0.0 | 0.4±0.1 | 0.5±0.1 |
| Hexadecanoic acid | 94.8±10.8 | **56.1±5.9** | 97.1±9.2 | **71.3**±3.5 | 95.5±7.6 | 87.9±9.2 |
| Hexadecanoic acid, 1-[[oxy]methyl]-1,2-ethanediyl | 2.9±0.2 | **ND** | 3.4±0.1 | **ND** | 2.7±0.3 | **ND** |
| Nonanoic acid | 7.6±2.0 | **5.0±0.8** | 7.8±1.6 | 5.3±1.0 | 4.3±0.6 | **1.6**±0.5 |
| Octacosanoic acid | 0.5±0.1 | **0.3±0.1** | 0.8±0.1 | 0.5±0.1 | 0.7±0.0 | 0.4±0.1 |
| Octadecanoic acid | 33.0±2.3 | 27.8±3.0 | 32.9±2.0 | 33.3±2.6 | 30.5±2.5 | 28.5±3.1 |
| Oleanitrile | 1.6±0.3 | 1.7±0.4 | 1.4±0.4 | **ND** | ND | ND |
| Pentacosanoic acid | 0.5±0.1 | 0.3±0.0 | 0.7±0.1 | **0.3**±0.1 | 0.3±0.0 | 0.3±0.0 |
| Pentadecanoic acid | 1.6±0.3 | **ND** | 1.7±0.0 | **ND** | 1.8±0.2 | **ND** |
| Tetracosanoic acid | 4.0±0.7 | **ND** | 3.7±0.4 | **ND** | 2.8±0.6 | ND |
| Triacontanoic acid | 0.9±0.1 | **0.5±0.1** | 1.1±0.1 | **0.5**±0.0 | 1.0±0.2 | 0.5±0.1 |
| Tricosanoic acid | 0.4±0.1 | 0.5±0.0 | 0.4±0.2 | 0.5±0.1 | 0.4±0.2 | 0.4±0.1 |
| ***Nitrogenous metabolites*** | | | | | | |
| 2-Methylserine | 2.5±0.2 | 2.8±0.2 | 1.2±0.1 | **2.7**±0.2 | 1.6±0.2 | **4.7**±0.6 |
| 2-Aminobutyric acid | 1.3±0.1 | 1.5±0.1 | 1.7±0.3 | **3.9**±0.1 | 1.7±0.2 | **5.5**±0.2 |
| 2-Aminoethylphosphate | 3.7±0.9 | 7.6±2.0 | 0.8±0.1 | **5.3**±0.3 | ND | **3.7**±0.8 |
| 5-Methylthioadenosine | 10.6±2.4 | 5.7±1.0 | 8.3±0.6 | **4.1**±0.5 | 6.9±0.2 | **4.9**±0.2 |
| Adenine | 3.6±0.5 | 2.8±0.4 | 2.8±0.3 | 2.7±0.7 | 3.1±0.4 | 2.6±0.3 |
| Adenosine | 14.0±2.4 | **46.2±8.1** | 16.1±1.5 | **48.5**±4.3 | 17.9±1.0 | **44.9**±6.5 |
| Adenosine-5-P | 32.6±3.8 | 23.5±3.1 | 28.0±1.6 | 25.6±2.2 | 16.2±1.9 | **22.7**±1.2 |
| Alanine | 235.2±11.5 | **693.1±97.3** | 207.2±27.0 | **747.8**±80.6 | 345.3±33.0 | **1004.4**±89.3 |
| Amino isobutyric acid | 5.3±0.9 | 10.0±2.2 | 2.6±0.3 | **10.7**±1.6 | 3.2±0.4 | **15.2**±0.8 |
| Aminomalonic acid | 1.0±0.2 | 1.3±0.4 | 0.4±0.1 | **1.4**±0.2 | 0.7±0.1 | 1.2±0.2 |
| Asparagine | 40.3±2.0 | 39.4±2.6 | 37.6±1.2 | 27.8±2.0 | 35.2±4.3 | 21.9±1.9 |
| Aspartic acid | 157.5±18.6 | **91.7±6.3** | 99.1±3.0 | 89.0±3.0 | 93.0±7.9 | 127.0±11.7 |
| β-Alanine | 14.5±2.5 | **67.1±6.2** | 3.4±0.2 | **57.0**±5.4 | 3.9±0.5 | **36.5**±1.3 |
| Butylamine | 0.7±0.1 | **0.2±0.0** | 0.6±0.1 | **0.2**±0.0 | 1.0±0.2 | **0.2**±0.1 |
| Cadaverine | 0.5±0.1 | **19.9±2.2** | 0.5±0.0 | **19.5**±2.2 | 0.5±0.1 | **18.0**±1.7 |
| Cysteine | 4.0±1.0 | 5.2±0.6 | 2.2±0.3 | **7.5**±0.3 | 9.0±1.0 | 7.0±0.6 |
| Cytosine | 0.2±0.1 | 0.2±0.0 | 0.3±0.1 | 0.2±0.0 | 0.2±0.0 | 0.2±0.1 |
| Ethanolamine | 184.8±10.0 | **334.7±51.3** | 165.6±35.6 | **272.9**±16.1 | 159.4±6.9 | **280.3**±46.3 |
| GABA (γ-aminobutyric acid) | 154.2±24.4 | **405.1±13.4** | 120.1±10.9 | **455.8**±8.8 | 53.2±6.6 | **446.0**±33.9 |
| Glutamic acid | 430.6±68.4 | **152.6±32.5** | 235.0±11.5 | **129.8**±39.0 | 242.4±31.6 | **136.0**±17.5 |
| Glutamine | 38.5±7.8 | **15.7±3.4** | 26.9±3.7 | **12.9**±3.2 | 163.5±37.6 | 13.8±3.2 |
| Glycine | 176.4±33.9 | **344.7±58.8** | 188.7±3.7 | 203.2±42.6 | 208.1±34.0 | 296.6±37.7 |
| Guanine | 0.2±0.0 | **ND** | 0.2±0.0 | **ND** | 0.2±0.0 | ND |
| Histidine | 38.0±1.4 | **1.7±0.3** | 28.3±3.1 | **1.7**±0.2 | 30.4±3.0 | **2.0**±0.1 |
| Homocysteine | 0.4±0.1 | 0.3±0.0 | 0.5±0.0 | **0.1**±0.0 | 0.4±0.1 | **0.1**±0.0 |
| Homoserine | 5.2±0.2 | 4.3±0.4 | 3.5±0.3 | **2.3**±0.2 | 2.9±0.1 | 3.3±0.5 |
| Hydroxylamine | 5.5±0.2 | **13.8±1.4** | 3.5±0.4 | **9.9**±2.3 | 4.1±0.8 | **11.2**±3.3 |
| Inosine | 2.4±0.3 | **ND** | 4.7±0.2 | **ND** | 1.4±0.3 | ND |
| Isoleucine | 34.5±2.2 | 25.9±1.8 | 30.4±1.9 | 28.0±2.8 | 83.7±10.2 | 78.7±8.4 |
| Leucine | 83.5±2.4 | **34.5±7.9** | 64.6±5.7 | **47.1**±0.4 | 257.8±30.6 | **76.5**±6.5 |
| Lysine | 39.8±5.4 | **16.4±3.3** | 34.1±3.7 | 27.1±2.0 | 60.4±5.4 | **21.8**±1.3 |
| Methionine | 13.4±3.2 | **4.6±0.3** | 11.9±0.2 | **3.9**±0.6 | 11.6±1.8 | **3.5**±0.2 |
| N-Acetylglutamic acid | 15.7±3.4 | **4.7±0.4** | 24.0±1.3 | **6.2**±0.6 | 21.6±2.5 | **6.1**±0.4 |
| N-Acetyl-serine | 3.6±0.5 | 4.4±0.2 | 5.4±0.8 | 3.7±0.7 | 8.0±0.9 | **4.1**±0.6 |
| Ornithine | 1.4±0.2 | **0.3±0.1** | 0.8±0.1 | 0.5±0.1 | 0.5±0.0 | 0.5±0.1 |
| Phenylalanine | 36.3±3.3 | **17.1±2.8** | 32.6±0.8 | **17.5**±2.7 | 109.9±13.2 | **20.8**±3.1 |
| Pipecolic acid | 1.2±0.0 | 2.5±0.5 | 1.6±0.2 | **2.6**±0.1 | 1.5±0.2 | **2.6**±0.3 |
| Proline | 48.1±6.4 | **28.6±7.9** | 25.8±3.2 | 23.3±3.0 | 30.3±2.8 | 33.0±2.1 |
| Putrescine | 18.4±2.1 | **691.8±13.1** | 15.8±4.2 | **742.1**±10.3 | 41.0±3.1 | **771.0**±39.0 |
| Pyroglutamic acid | 82.2±4.5 | **44.4±2.7** | 78.3±1.8 | **59.4**±0.4 | 283.8±45.1 | **68.8**±7.6 |
| Serine | 248.3±23.3 | **357.0±28.6** | 116.0±15.4 | **272.5**±10.2 | 179.6±32.7 | 155.3±39.1 |
| Spermidine | 19.1±1.7 | 17.9±2.9 | 13.9±4.9 | 21.3±2.1 | 14.4±2.7 | 20.8±2.2 |
| Suberyl glycine | 1.8±0.2 | **2.9±0.2** | 0.5±0.0 | **2.9**±0.3 | 0.6±0.1 | **2.4**±0.4 |
| Threonine | 231.0±32.4 | **95.3±4.7** | 189.1±10.3 | **107.2**±4.6 | 140.2±49.5 | 97.3±7.8 |
| Thymine | 0.3±0.1 | **0.1±0.0** | 0.3±0.0 | **0.1**±0.0 | 0.1±0.0 | 0.1±0.0 |
| Tryptophan | 57.0±3.4 | **6.3±0.7** | 37.0±9.5 | **7.8**±0.3 | 47.1±3.2 | **5.3**±0.3 |
| Tyrosine | 37.9±3.1 | **2.6±0.3** | 35.1±2.6 | **3.9**±0.3 | 43.2±4.5 | **5.2**±0.8 |
| Uracil | 2.8±0.1 | **18.1±2.0** | 2.3±0.3 | **15.9**±2.4 | 2.4±0.1 | **9.7**±0.5 |
| Urea | 1.6±0.2 | **5.0±0.6** | 1.6±0.1 | **9.2**±0.9 | 1.6±0.1 | **7.8**±0.6 |
| Uric acid | 3.3±0.4 | 2.2±0.4 | 1.6±0.1 | 2.1±0.2 | 1.6±0.1 | 1.4±0.2 |
| Uridine | 11.8±1.7 | **23.1±2.5** | 17.6±2.7 | 22.6±2.3 | 17.3±1.3 | 22.2±0.5 |
| Valine | 63.0±5.0 | **36.0±0.8** | 59.8±3.1 | **41.5**±1.5 | 70.3±5.0 | **45.0**±7.8 |
| ***Organic acids*** |  |  |  |  |  |  |
| 1-Aminocyclopropanecarboxylic acid | 30.6±2.3 | **44.6**±4.5 | 46.2±4.3 | **85.8**±7.1 | 29.2±3.5 | **102.9**±14.7 |
| 2,4,5-Trihydroxypentanoic acid | 1.8±0.2 | 1.8±0.2 | 1.4±0.4 | 1.6±0.3 | 1.0±0.1 | 1.3±0.3 |
| 2,4-Hydroxybutanoic acid | 2.5±0.2 | 2.3±0.4 | 2.4±0.3 | 1.7±0.3 | 1.0±0.3 | 1.7±0.5 |
| 2-Indole carboxylic acid | 7.7±0.7 | **29.7±3.1** | 6.9±1.2 | **35.5**±3.0 | 7.6±0.9 | **33.0**±2.3 |
| 2-Keto-gluconic acid | 110.9±5.4 | **56.1±2.7** | 174.0±17.1 | **56.0**±6.9 | 160.8±32.1 | **62.0**±10.2 |
| 2-Methylbenzoic acid | 0.6±0.1 | 0.6±0.2 | 0.8±0.2 | 0.5±0.0 | 1.0±0.1 | 0.7±0.2 |
| 3,4-Dihydroxybutanoic acid | 0.4±0.1 | 0.3±0.1 | 0.4±0.1 | 0.4±0.1 | 0.5±0.1 | 0.4±0.0 |
| 3-Hydroxymethylglutaric acid | 290.3±63.2 | **10.0±1.5** | 270.0±19.2 | **8.4**±0.5 | 66.4±3.8 | **12.1**±1.8 |
| 4-Hydroxybutanoic acid | 1.9±0.3 | 2.9±0.5 | 1.9±0.1 | 2.0±0.3 | 2.1±0.1 | 2.0±0.3 |
| Aconitic acid | 3.7±0.2 | **ND** | 7.3±1.1 | **ND** | 6.2±1.0 | **ND** |
| α-Ketoglutaric acid | 4.0±0.1 | **1.8±0.4** | 3.4±0.3 | 2.6±0.2 | 6.0±1.0 | 3.8±0.1 |
| Arabinonic acid, lactone | 1.1±0.3 | **3.3±0.2** | 1.7±0.3 | **3.2**±0.2 | 1.2±0.2 | **3.0**±0.1 |
| Ascorbic acid | 73.3±4.7 | 66.7±1.8 | 66.5±3.3 | **45.0**±2.2 | 17.9±1.2 | 14.9±0.8 |
| Benzoic acid | 0.7±0.1 | **ND** | 0.7±0.1 | **ND** | 0.6±0.2 | ND |
| Citric acid | 141.1±9.5 | **20.0±2.9** | 125.4±10.5 | **19.5**±1.8 | 77.4±5.4 | **18.8**±2.0 |
| Dehydroascorbic acid | 13.8±2.7 | 12.2±2.4 | 22.6±2.6 | 27.9±2.0 | 1.9±0.5 | 2.7±0.2 |
| Fumaric acid | 13.7±0.9 | 19.8±2.6 | 28.4±4.1 | 32.6±1.3 | 39.3±8.9 | 28.9±3.0 |
| Galactaric acid | 1.3±0.3 | **3.6±0.5** | 1.9±0.2 | **6.9**±0.5 | 1.8±0.3 | **5.7**±1.4 |
| Galactonic acid | 6.8±0.6 | **15.7±3.1** | 7.1±0.4 | **15.1**±3.8 | 6.3±1.0 | **16.3**±1.3 |
| Glucaric acid | 1.6±0.7 | **3.3±0.6** | 2.2±0.3 | 3.2±0.3 | 1.5±0.4 | **3.5**±0.2 |
| Gluconic acid | 2.7±0.1 | **4.5±0.6** | 2.3±0.2 | **4.6**±0.4 | 12.2±2.1 | **6.8**±1.4 |
| Gluconic acid, lactone | 42.6±2.6 | **28.9±1.7** | 45.7±7.7 | 27.7±3.1 | 23.1±2.6 | 33.6±2.9 |
| Glyceric acid | 15.3±0.7 | **24.6±1.3** | 25.3±1.6 | 26.9±1.8 | 34.6±3.3 | **21.2**±2.0 |
| Glycolic acid | 2.6±0.4 | **5.2±0.1** | 3.4±0.3 | 5.0±0.4 | 3.3±0.4 | 4.7±0.3 |
| Gluonic acid | 4.8±0.4 | 6.1±0.5 | 3.4±0.1 | 4.5±0.4 | 3.8±0.4 | 5.1±0.4 |
| Hexanoic acid | 0.3±0.1 | 0.3±0.0 | 0.2±0.1 | 0.4±0.1 | 0.1±0.1 | 0.2±0.0 |
| Hydroxymalonic acid | 0.2±0.0 | **ND** | 0.3±0.0 | **ND** | 0.2±0.0 | ND |
| Lactic acid | 139.8±10.7 | **75.8±4.9** | 179.9±41.0 | 112.0±7.0 | 273.4±36.3 | **151.4**±35.9 |
| Maleic acid | 0.2±0.0 | 0.2±0.1 | 0.2±0.0 | 0.3±0.0 | 0.2±0.1 | 0.3±0.1 |
| Malic acid | 1056.2±83.1 | **422.9±26.5** | 1798.7±73.2 | **528.5**±19.4 | 1891.3±94.9 | **529.9**±38.6 |
| Malonic acid | 0.2±0.0 | **0.9±0.2** | 0.2±0.1 | **0.7**±0.1 | 0.3±0.1 | **1.9**±0.2 |
| Nicotinic acid | 6.8±1.0 | 4.7±0.8 | 6.0±0.3 | 4.1±0.2 | 3.9±0.6 | 3.7±0.4 |
| Orotic acid | 0.3±0.1 | **ND** | 0.2±0.0 | **ND** | 0.1±0.0 | ND |
| Oxalic acid | 5.9±0.6 | 6.4±1.0 | 10.2±1.2 | **6.3**±0.3 | 5.2±0.6 | 6.1±0.5 |
| Pyruvic acid | 5.7±1.0 | **9.7±0.3** | 10.2±1.1 | **21.5**±0.8 | 14.3±4.0 | 12.4±0.2 |
| Quinic acid | 2.6±0.1 | **4.9±0.7** | 6.2±1.3 | 7.7±0.3 | 12.1±2.9 | 14.0±2.3 |
| Quinolinic acid | 191.9±21.2 | **3.5±0.4** | 210.4±27.4 | **4.9**±0.8 | 105.0±11.9 | **10.7**±2.5 |
| Ribonic acid | 31.6±4.5 | 26.0±2.2 | 28.6±4.1 | 25.1±1.1 | 23.0±2.7 | 28.5±1.0 |
| Shikimic acid | 4.9±0.5 | **2.8±0.3** | 12.2±2.0 | **2.4**±0.2 | 10.5±1.0 | **1.6**±0.1 |
| Succinic acid | 77.2±12.4 | **237.0±24.0** | 74.5±3.6 | **262.6**±28.8 | 124.3±29.2 | **261.8**±19.6 |
| Tartaric acid | 2.9±0.3 | 4.5±0.2 | 6.0±1.5 | 3.8±0.1 | 5.2±0.3 | 7.0±0.8 |
| Threonic acid | 20.6±1.9 | **37.6±0.6** | 36.0±2.8 | 44.1±5.2 | 59.6±6.1 | 55.5±2.1 |
| Threonic acid, lactone | 1.1±0.3 | 1.3±0.3 | 1.0±0.2 | 1.5±0.2 | 1.1±0.3 | 1.3±0.2 |
| Xylonic acid, lactone | 10.9±2.0 | **3.2±0.6** | 10.0±1.4 | **3.4**±0.3 | 6.5±0.5 | **1.9**±0.2 |
| ***Sterols*** |  |  |  |  |  |  |
| Campesterol | 3.2±0.4 | **0.4**±0.1 | 3.8±0.1 | **0.4**±0.1 | 2.7±0.3 | **0.3**±0.1 |
| Sitosterol | 51.9±8.0 | **26.4±0.4** | 53.2±10.0 | 34.5±1.6 | 41.7±4.1 | 31.0±1.0 |
| Tocopherol (Vitamine E) | 4.2±0.7 | **0.9±0.1** | 4.2±0.7 | **1.2**±0.0 | 3.0±0.7 | **1.5**±0.3 |
| ***Sugars*** |  |  |  |  |  |  |
| 1,6-Anhydroglucose | 7.8±0.7 | **3.7**±0.3 | 8.1±0.2 | **2.6**±0.3 | 5.2±0.2 | **2.6**±0.4 |
| 1-Ethylglucopyranoside | 512.4±54.7 | 632.8±8.9 | 502.5±29.5 | 406.0±32.0 | 177.9±1.1 | **381.4**±22.7 |
| 1-Methyl-α-D-galactopyranoside | 3.4±0.3 | **15.3±3.1** | 2.9±0.1 | **16.1**±3.6 | 5.8±1.4 | **11.6**±1.2 |
| 1-Methyl-β-D-galactopyranoside | 4.9±1.0 | **28.5±3.5** | 7.5±1.9 | **60.6**±9.7 | 11.9±1.9 | **39.2**±4.3 |
| 2-O-Glycerol-α-D-galactopyranoside | 6.2±0.4 | **31.4±4.8** | 5.6±0.8 | **34.8**±2.2 | 19.1±2.3 | 20.9±2.6 |
| 2-O-Glycerol-β-D-galactopyranoside | 24.9±0.4 | **67.2±2.9** | 34.4±2.5 | **69.4**±7.3 | 32.0±1.5 | **70.2**±6.4 |
| 6-Deoxymannopyranose | 4.1±0.3 | **5.2±0.3** | 3.4±0.2 | 3.8±0.5 | 1.9±0.1 | **4.0**±0.2 |
| Arabinose | 3.8±0.2 | 4.6±0.3 | 14.4±1.4 | **4.6**±0.8 | 15.2±0.1 | **9.7**±0.4 |
| Digalactosylglycerol | 5.0±0.9 | **20.8±0.6** | 7.4±1.1 | **49.2**±8.2 | 13.8±3.5 | 23.7±4.0 |
| Fructofuranoside | 857.3±21.3 | **123.8±11.9** | 614.8±35.0 | **97.4**±13.1 | 481.9±49.5 | **159.4**±25.8 |
| Fructose | 1083.5±257.7 | **5257.7±361.8** | 983.4±238.1 | **5546.1**±261.6 | 1418.3±249.7 | **6452.8**±275.1 |
| Fructose-6-P | 19.4±3.6 | 14.3±1.1 | 9.6±0.8 | 14.5±0.8 | 3.2±0.2 | **0.5**±0.1 |
| Galactofuranose | 3.6±0.2 | **19.0±2.8** | 1.6±0.4 | **15.1**±1.0 | 0.5±0.1 | **20.2**±2.8 |
| Galactosamine | ND | **2.2±0.2** | ND | **2.7**±0.3 | ND | **2.8**±0.3 |
| Galactose | 223.3±39.8 | **947.4±32.0** | 240.5±36.2 | **1301.0**±78.3 | 276.5±18.8 | **1366.7**±95.0 |
| Gentiobiose | 91.3±5.7 | **37.5±1.4** | 80.8±4.4 | **39.4**±8.8 | 74.1±3.0 | **42.8**±2.4 |
| Glucoheptulose | 8.0±0.7 | **10.6±1.6** | 9.1±0.9 | 12.9±3.9 | 11.1±1.2 | 14.8±3.9 |
| Glucopyranose | 523.5±28.3 | **2174.9±163.2** | 663.7±41.7 | **1636.3**±255.5 | 554.6±32.8 | **1468.9**±248.3 |
| Glucosamine | ND | **14.3±2.2** | ND | **10.6**±1.4 | ND | **16.5**±3.0 |
| Glucose | 1211.0±163.7 | **2720.8±199.0** | 1251.8±141.9 | **2948.8**±269.8 | 1447.5±78.0 | **3567.0**±274.2 |
| Glucose-1-P | 19.7±3.4 | 24.9±2.5 | 22.9±1.9 | 34.8±0.9 | 9.4±1.2 | **44.0**±4.1 |
| Glucose-6-P | 92.9±4.8 | **23.0±0.9** | 125.3±23.8 | **24.3**±3.3 | 18.5±1.1 | 18.7±2.8 |
| Isomaltose | 3.1±0.5 | 3.0±0.3 | 19.1±3.8 | **3.2**±0.4 | 16.9±2.3 | **6.8**±0.6 |
| Maltose | 13.6±3.9 | 8.9±0.8 | 18.5±3.3 | **7.8**±0.8 | 15.7±2.7 | **7.1**±0.7 |
| Mannose | 4.1±0.8 | **50.0±1.4** | 47.7±4.4 | 50.0±2.8 | 51.6±2.1 | 58.3±4.3 |
| Mannose-6-P | 3.0±0.2 | **1.4±0.1** | 3.6±0.3 | 2.2±0.3 | 3.2±0.1 | **1.9**±0.1 |
| Melibiose | 26.8±2.6 | **12.5±1.1** | 26.4±3.5 | **14.3**±1.7 | 27.2±2.7 | **11.0**±1.0 |
| N-Acetyl glucosamine | 1.8±0.5 | 1.4±0.2 | 1.8±0.1 | 1.6±0.2 | 1.5±0.4 | 1.6±0.2 |
| N-Acetylglucosylamine | 2.9±0.7 | **ND** | 2.4±0.3 | **ND** | 1.4±0.1 | ND |
| Rhamnose | 4.5±1.6 | 3.7±0.2 | 3.7±0.3 | 3.6±0.2 | 1.9±0.4 | 2.4±0.0 |
| Ribose | 11.8±1.5 | **20.2±2.3** | 15.7±2.5 | **28.3**±3.7 | 25.7±4.4 | 15.3±2.2 |
| Sedoheptulose | 37.8±3.6 | 45.8±3.4 | 39.6±3.2 | 56.0±4.7 | 35.0±3.7 | **62.3**±4.1 |
| Sorbopyranose | 488.5±45.0 | **1716.4±183.3** | 1034.8±163.7 | **2034.0**±276.0 | 569.9±62.3 | **1926.1**±180.0 |
| Sorbose | 812.5±51.3 | **3246.0±224.6** | 979.2±67.6 | **3081.7**±47.0 | 1223.4±151.3 | **3490.5**±293.1 |
| Sucrose | 4591.7±50.8 | **2203.0±68.5** | 4611.1±387.4 | **2930.1**±107.2 | 4779.1±435.8 | **3224.1**±447.2 |
| Sucrose-6-P | 2.5±0.6 | 1.7±0.2 | 3.2±0.7 | **1.3**±0.1 | 0.5±0.1 | **0.2**±0.0 |
| Galactopyranose | 1181.9±286.7 | **2286.4±222.6** | 1068.9±78.1 | **2499.2**±173.6 | 966.6±28.6 | **2525.6**±429.7 |
| Trehalose | 40.1±8.8 | 40.1±4.2 | 53.2±1.8 | 40.1±2.1 | 41.4±1.6 | 40.6±1.1 |
| Trehalose-6-P | 2.6±0.6 | 2.2±0.2 | 3.3±0.2 | **1.6**±0.1 | 5.8±0.7 | **1.9**±0.3 |
| **Other metabolites** |  |  |  |  |  |  |
| 2,4,6-Tri-tert.-butylbenzenethiol | 0.4±0.1 | 0.4±0.0 | 0.6±0.1 | 0.5±0.1 | 0.5±0.0 | 0.5±0.1 |
| 3,4-Hydroxy-2(3H)-Furanone | 1.6±0.2 | 1.6±0.1 | 1.5±0.4 | 1.5±0.2 | 3.8±0.8 | **1.5±0.2** |
| 3-phosphoglycerate | 6.1±0.2 | 3.6±0.5 | 3.1±0.4 | 2.7±0.2 | 0.3±0.1 | **1.4±0.1** |
| β-Amyrin | 0.5±0.1 | 0.4±0.1 | 1.3±0.3 | 1.5±0.1 | 1.1±0.2 | 1.7±0.3 |
| Ethyleneglycol | 1.7±0.4 | 1.9±0.3 | 1.4±0.2 | **1.8±0.3** | 1.5±0.2 | **2.2±0.3** |
| Monomethylphosphate | 1722.6±91.8 | 1611.2±93.9 | 1428.1±61.8 | **1282.8±108.4** | 346.5±29.7 | **1354.0±216.2** |
| Pantothenic acid – Vitamin B5 | 0.4±0.1 | **5.6±0.5** | 1.5±0.5 | **6.6±1.9** | 0.2±0.0 | **4.6±0.7** |
| 3-[(2,2-dimethylpropylidene) amino]propylphosphate | 30.8±2.3 | 45.6±4.2 | 13.8±0.4 | **22.4±2.1** | 5.1±0.3 | **16.5±3.1** |
| Phosphate | 3250.2±157.3 | 2856.1±138.7 | 2421.8±117.2 | 2412.2±291.4 | 990.2±71.7 | **1483.9±330.8** |
| Ribofuranosyl-2(1H)-Pyrimidinone | 5.0±0.4 | 2.7±0.8 | 7.5±1.4 | **39.7±3.0** | 22.1±1.2 | **33.3±2.7** |
